# Chiral amines in the asymmetric synthesis of axially chiral allenes through catalysis and covalent chirality transfer

**DOI:** 10.1039/d6ra09750b

**Published:** 2026-11-11

**Authors:** Laxman M. Alakonda, Manal A. Nael, Khaled M. Elokely

**Affiliations:** a Division of Pharmaceutical Sciences, School of Pharmacy, University of Wyoming Laramie Wyoming 82071 USA kelokely@uwyo.edu +(307) 766-6136

## Abstract

Axially chiral allenes are useful motifs and intermediates in stereoselective synthesis. Chiral amines provide several routes to these structures, but their operational roles differ. An amine may act as a regenerated organocatalyst, cooperate with a metal catalyst, or serve as a stoichiometric covalent mediator that transfers stereochemical information through a propargylamine. This review compares these roles using amine loading, substrate activation, stereochemical evidence and regeneration as organizing criteria. Noncovalent additions, isomerizations and proton-transfer reactions are considered alongside enamine and dienamine catalysis, stereoselective functionalization of pre-existing allenes, and cooperative palladium–amine and gold–squaramide chemistry. Metal-assisted rearrangements of chiral propargylamines provide complementary access to allenes through covalent chirality transfer. Quantitative examples distinguish matched yields and selectivities from scope maxima, and separate reaction performance from mediator recovery after chemical reduction. The literature demonstrates both catalytic axis construction and catalytic control of axial and central stereochemistry. A remaining challenge is to extend amine turnover to broadly modular alkyne–carbonyl–propargylamine sequences while closing the hydrogen-transfer and regeneration balance. Progress will require substrate-specific mechanistic evidence, reproducible stereochemical analysis and quantitative assessment of temperature, scale and reagent demand.

## Abstract 2

Chiral amines control allenic axial chirality through catalytic activation or covalent relays; comparing turnover and stereochemical evidence reveals where each strategy succeeds.

## Graphical abstract

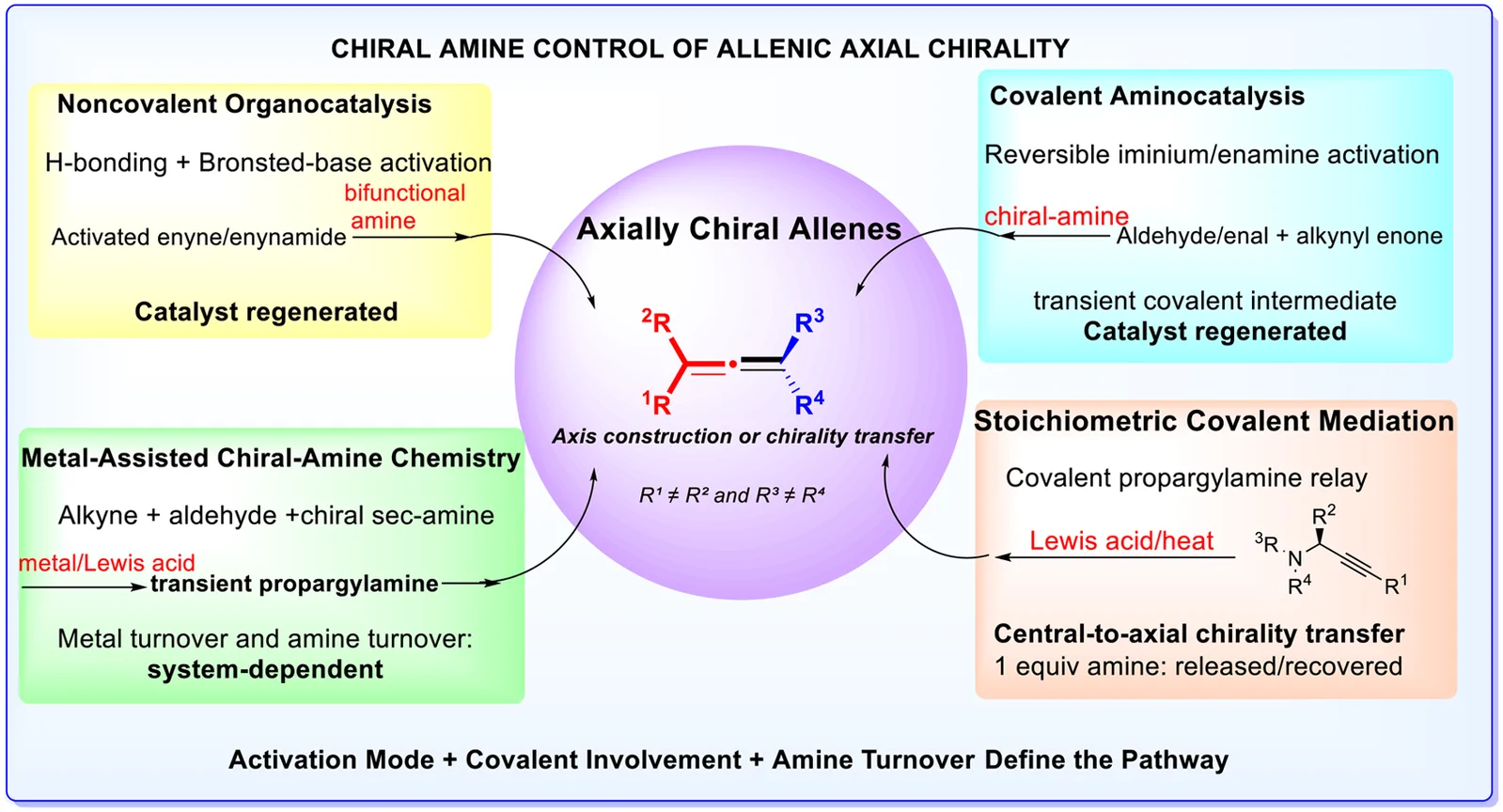

## 1 Introduction

Allenes contain two cumulated carbon–carbon double bonds whose π systems occupy orthogonal planes. When the two substituents at each terminus are constitutionally different, this geometry creates a stereogenic axis and permits the isolation of enantiomeric allenes when racemization is sufficiently slow. The resulting combination of compact three-dimensional shape, differentiated termini, and orthogonal reactivity has made enantioenriched allenes useful as natural-product motifs, ligands, functional materials, and intermediates from which axial information can be transferred into one or more stereogenic centers.^1–5^

The synthesis of optically active allenes has evolved from resolution and substrate-controlled chirality transfer to catalytic asymmetric construction of the allene axis.^2–8^ Classical approaches frequently begin with an enantioenriched propargylic alcohol, ester, amine, or related leaving-group derivative and exploit a stereospecific substitution or rearrangement. Modern transition-metal catalysis can generate the allene axis directly from achiral or racemic precursors, while organocatalysis has enabled isomerization, addition, and functionalization pathways under complementary conditions.^6–10^ Despite this progress, direct and modular access to configurationally defined allenes from simple alkynes and carbonyl compounds remains challenging.

Chiral amines occupy an unusual position in this field. Depending on structure and stoichiometry, the amine can act as a nucleophilic Lewis-base catalyst, a Brønsted base, an ion-pairing or hydrogen-bonding catalyst, a ligand or reaction partner in cooperative metal catalysis, or a temporary covalent template that forms a diastereomerically enriched propargylamine. These pathways are mechanistically distinct, and terminology has varied across the literature. Accordingly, this review considers whether the amine is regenerated, how much is used, which step establishes stereochemistry, and whether the measured enantioenrichment arises during allene formation or through resolution of an already formed allene.

Earlier reviews address complementary questions. Chu, Zhang and Wang survey catalytic asymmetric allene synthesis broadly,^6*b*^ whereas Li and co-workers organize asymmetric 1,4-difunctionalization of 1,3-enynes into radical and non-radical strategies.^11^ Woldegiorgis and co-workers focus on chiral-phosphoric-acid-catalyzed access to axially chiral allenes and styrenes.^10^ Other accounts cover organocatalytic allene synthesis^9^ and heteroatom-substituted allenes.^12^ These catalyst- or transformation-centered perspectives provide essential context. Here, the deliberately focused amine scope allows catalytic activation and stoichiometric covalent chirality transfer to be assessed within one operational framework, rather than treating them as interchangeable routes.

The contribution of this review is therefore an integrated assessment of three connected questions: what establishes the allene configuration, whether the active amine is regenerated during productive reaction, and what the reported yield and selectivity actually measure. Amine loading, recovery and demonstrated reuse are evaluated alongside substrate activation, configuration assignment and the strength of mechanistic evidence. This comparison identifies both complementary synthetic capabilities and the specific regeneration problem that separates a modular propargylamine relay from an amine-catalytic process. A comparison with earlier review scopes is provided in Section 6. The core coverage includes formation, functionalization or enrichment of an allenic axis under chiral-amine control; downstream consumption of that axis is discussed as synthetic utility (Scheme 1).

**Scheme 1:**
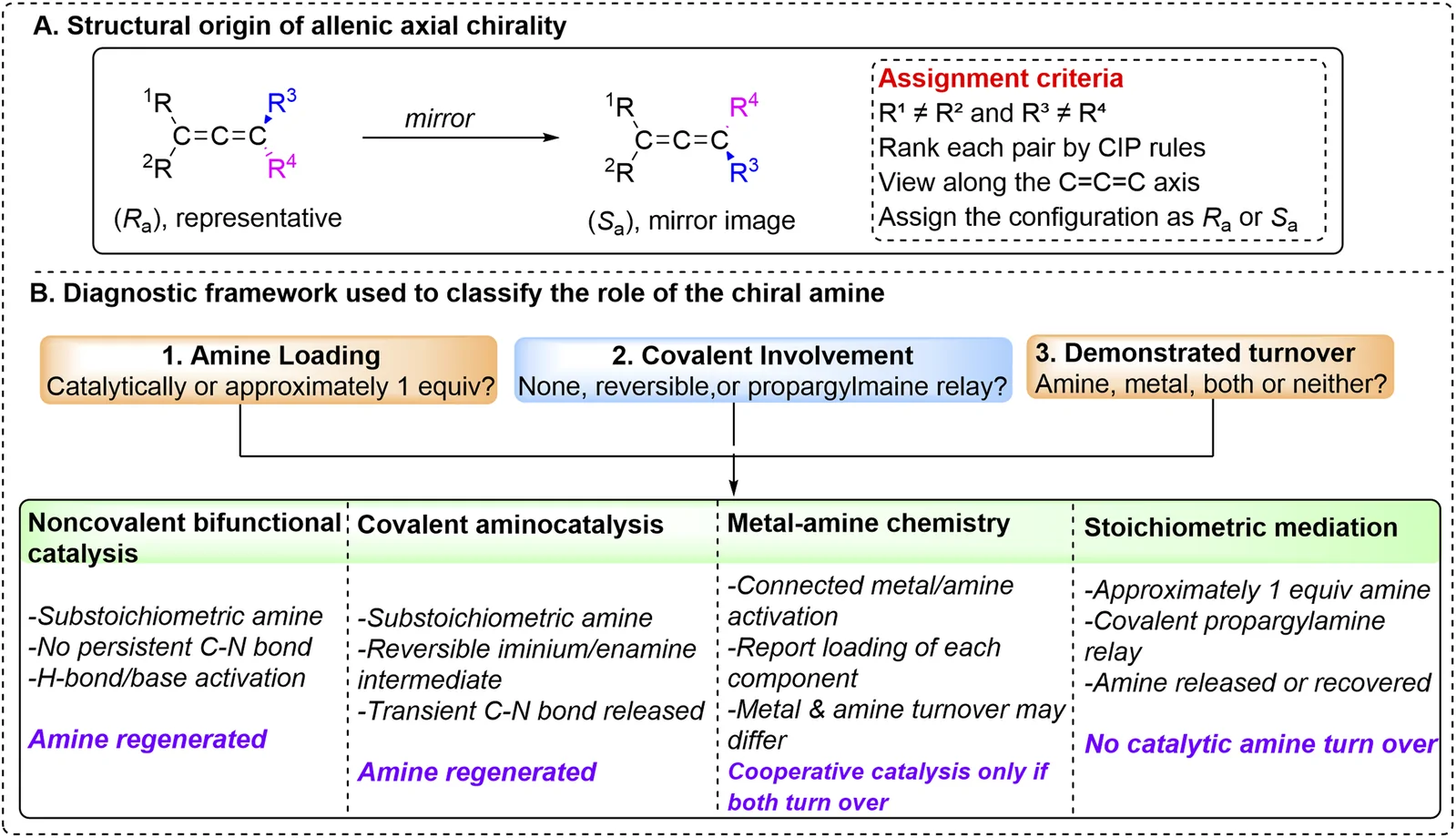
Structural basis of allenic axial chirality and operational classification of chiral-amine roles. (A) Absolute descriptors depend on the relative sequence-rule priorities at both allene termini. (B) Catalytic systems use substoichiometric amine with regeneration; stoichiometric covalent relays incorporate the chiral amine into a reaction intermediate. Metal-assisted chemistry can belong to either category depending on the demonstrated fate of the amine.

This critical narrative review covers selected literature published through September 2026. Literature was identified using SciFinder and Google Scholar, focusing on axially chiral allenes, chiral amines, organocatalysis, propargylamine rearrangements and chirality transfer. Primary studies were selected when a chiral amine-containing catalyst or covalent mediator controlled formation, functionalization or enrichment of an allenic axis and the reported transformation could be related to its stereochemical outcome. Noncovalent amine-containing catalysts and covalent primary- or secondary-amine catalysts are included. Reactions using only chiral phosphoric acids, chiral phosphines or achiral amines are outside the core scope but are discussed selectively as mechanistic or practical benchmarks. Transient allenes and downstream transformations are distinguished from isolated allene synthesis. Original articles, SI and published corrections are used to contextualize quantitative comparisons where available. The selection is purposive rather than exhaustive; no systematic-review protocol or bibliometric completeness is claimed.

## 2 Terminology and mechanistic foundations

### 2.1 Assignment and stability of allenic axial chirality

Absolute configuration is assigned by applying sequence rules at both allene termini and viewing along the cumulated axis. The descriptor depends on substituent priority, so drawings and assignments require rechecking when substituents change. Evidence is evaluated at two distinct levels: assignment of the product configuration and demonstration of its origin. Single-crystal X-ray analysis or a validated chemical correlation may establish configuration; chiral HPLC or GC normally establishes enantiomeric composition. Optical rotation, precursor dr, high product ee and agreement with a calculated transition state can support an interpretation but do not, individually, prove a stereospecific transfer pathway.

Configurational stability is equally important. Racemization can occur by thermal twisting, acid- or base-promoted pathways, reversible metal coordination, or addition–elimination sequences. An ee measured immediately after chromatography may therefore not describe the material under reaction or application conditions. Reports that compare time, temperature, solvent, and additives are particularly valuable, but remain uncommon. Future studies should report storage stability and the ee before and after representative derivatizations.

### 2.2 Catalysis, mediation, and auxiliary control

Chiral-amine organocatalysis requires regeneration of the active amine during productive reaction. A substoichiometric loading and product formation beyond the initial amine amount provide operational evidence for turnover, although they do not establish every elementary step. Noncovalent activation and reversible enamine, dienamine or iminium activation fall within this category. Cooperative catalysis requires productive involvement of both catalytic components. By contrast, an approximately stoichiometric amine incorporated into a propargylamine is a covalent mediator even when a metal catalyzes its subsequent rearrangement.

These categories describe a reaction, rather than a molecular scaffold in isolation. A prolinol-derived structure can act as a catalytic carbonyl activator in one system and as a covalent stereochemical reagent in another. The relevant experimental questions are which step establishes stereochemistry, whether the allene axis is formed or functionalized, and whether the starting amine is regenerated. The operational terminology used here supports comparison across methods without replacing the historical descriptions in the original reports.

Four outcomes are kept separate throughout: release of an amine-derived fragment by C–N cleavage; regeneration of the original active amine by a further chemical operation; isolated recovery of that amine; and demonstrated reuse in another reaction. None of the latter three, by itself, establishes *in situ* catalytic turnover. Recovery yields must state their denominator and work-up, while recycling claims require the yields and stereoselectivities of successive runs. Where repeated use or a closed amine balance is not documented in the evidence discussed, it is not inferred.

### 2.3 Formation and rearrangement of propargylamines

A recurring chiral-amine strategy joins a terminal alkyne, an aldehyde, and a secondary amine through an A3-type sequence. Metal acetylide formation is followed by addition to an iminium ion, producing a propargylamine. When the amine is chiral, diastereoselective carbon–carbon bond formation can establish a configurationally enriched propargylic center. Lewis-acid- or transition-metal-assisted rearrangement then converts this central stereochemical information into the allene axis (Scheme 2).

**Scheme 2:**
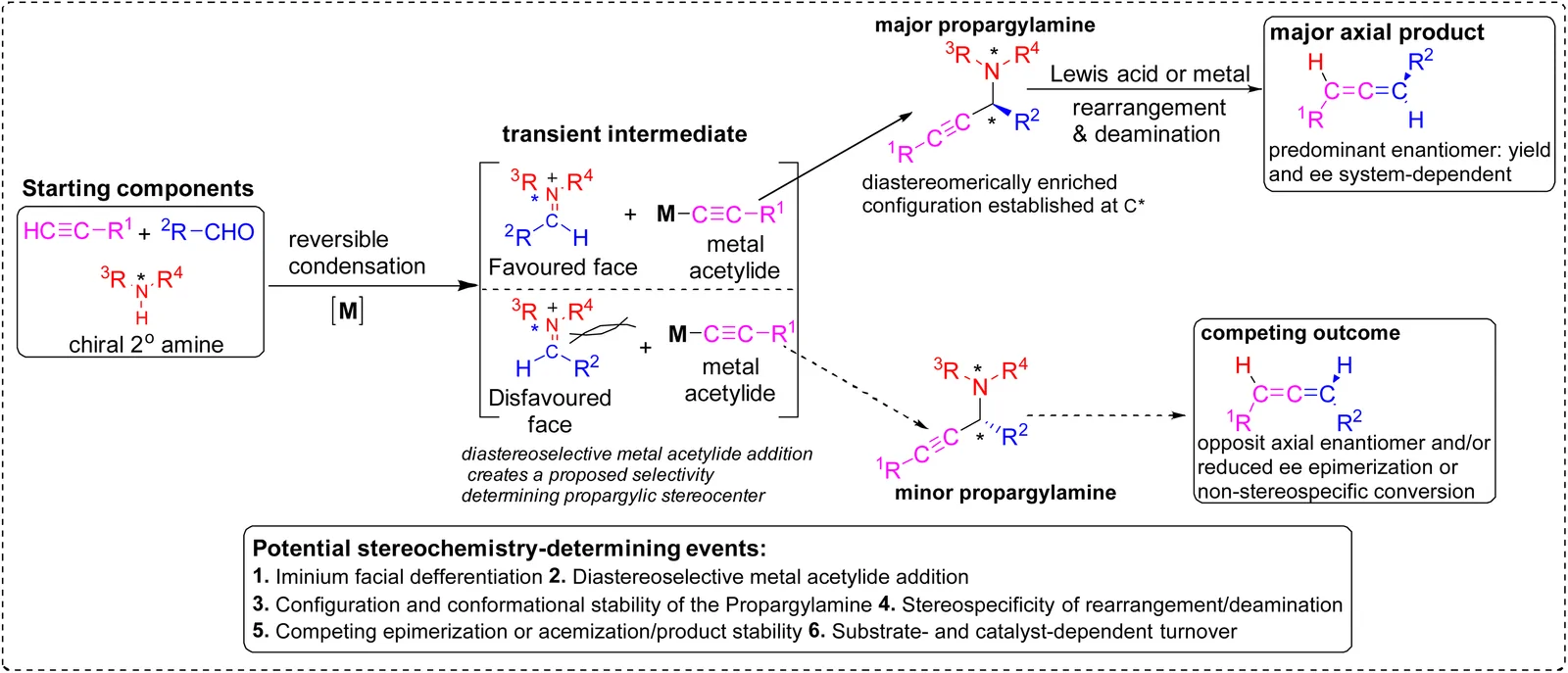
Generalized propargylamine relay linking precursor formation and central-to-axial chirality transfer. The sequence summarizes mechanistic proposals and experimentally observed transformations across different systems; it is not a universal catalytic cycle. The identity and oxidation state of the released amine-derived fragment, and any subsequent regeneration, depend on the reaction.

Metal coordination, conformational organization, hydrogen transfer and C–N bond cleavage form a common mechanistic proposal for the rearrangement. Independently prepared propargylamines have been converted to allenes,^13–17^ and calculations on the silver-mediated pathway favor stepwise hydride transfer and elimination.^18^ These findings support a covalent relay but do not establish one elementary sequence for every metal or substrate. Hydrogen transfer may produce an oxidized amine-derived imine; reduction is then a separate regeneration operation, as defined in Section 2.2.^17,19^

Demonstrating stereochemical transfer requires more than comparing a high precursor dr with a high product ee. Ideally, independently characterized precursor stereoisomers should be rearranged separately, with their conversion, product configuration, ee and material balance measured. Incomplete conversion, precursor epimerization, kinetic enrichment, product racemization and selective decomposition can otherwise give similar endpoint data. A study that has not assessed these alternatives supports a stereochemical correlation rather than their exclusion.

## 3 Catalytic chiral amine-controlled allene synthesis

Chiral amines control allene formation through several catalytic modes. Noncovalent catalysts can organize addition reactions or proton transfers, while secondary amines activate carbonyl compounds through reversible enamine or dienamine formation. Nucleophilic tertiary amines also enable stereoselective functionalization of an existing allene framework. These modes differ in the step that establishes the axis, the activation required in the substrates, and the process that regenerates the catalyst. The following discussion separates these functions and distinguishes isolated allene products from transient allene intermediates.^9^

### 3.1 Noncovalent chiral-amine catalysis

An early example is the cinchona-derived amino-urea-catalyzed syn-1,4-bromolactonization of conjugated (*Z*)-enynes reported by Zhang and co-workers.^20^ Intramolecular carboxylate addition and bromination form lactones bearing di-, tri-, or tetrasubstituted bromoallene units. The optimized study used 20 mol% catalyst and reported enantioselectivities up to 99% ee. The authors proposed bifunctional activation of the carboxylic acid and *N*-bromosuccinimide, but the detailed mode of brominating-agent activation remained tentative. This case belongs within allene synthesis because the isolated product retains the newly formed cumulene, despite the accompanying lactonization.

Inokuma and co-workers subsequently reported catalytic isomerization of racemic α-substituted alkynoates to enantioenriched trisubstituted allenoates using a bifunctional benzothiadiazine catalyst.^21^ The transformation was described as a dynamic kinetic resolution and furnished allenoates without substantial recovery of starting material. It establishes proton-transfer-mediated axis formation as an alternative to carbon–carbon bond construction. Its activated alkynoate substrates should be distinguished from the less activated alkynes employed in metal-assisted covalent-relay chemistry.

Qian, Yu, Zhang, and Sun developed the intermolecular addition of nitroalkanes to activated enynes 1 using a cinchona-derived tertiary amine–thiourea catalyst.^22^ The products are trisubstituted 2,3-allenoates 3A containing a nitroalkyl substituent (Scheme 3). The proposed addition/proton-transfer sequence uses the basic amine and hydrogen-bond donor to activate and organize the reacting partners; no stoichiometric propargylamine is required. This example therefore establishes catalytic access to an allenic axis from an activated enyne, although its electron-withdrawing ester and acidic nitroalkane partner are integral to the reported reaction design.

**Scheme 3:**
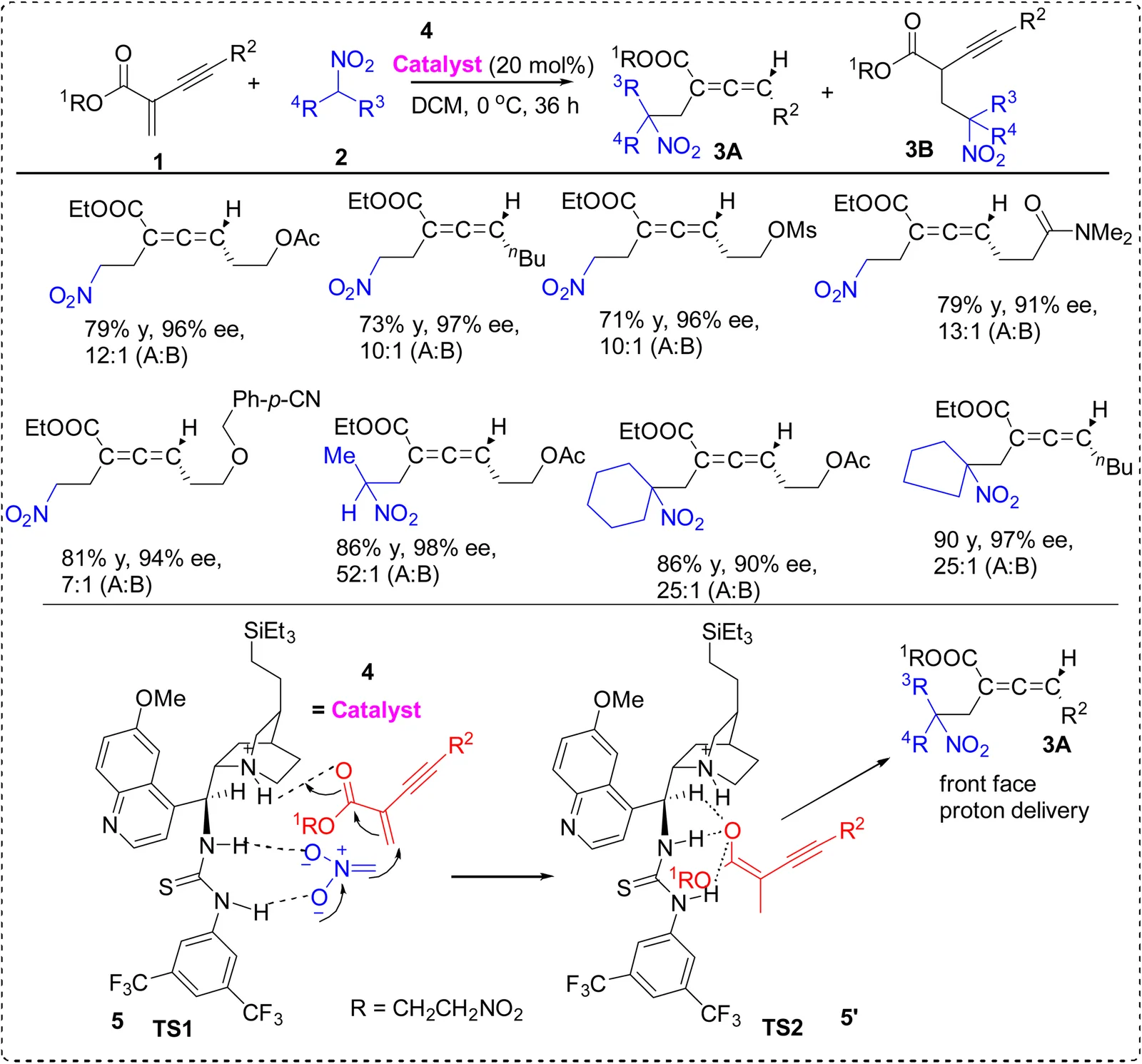
Bifunctional chiral-amine-catalyzed addition of nitroalkanes to activated enynes for the synthesis of 2,3-allenoates 3A.^22^

Ma and co-workers extended noncovalent catalysis to 2,3-allenamides 9 through reactions of hydrogen-bond-stabilized enynamides 6 with quinine-derived bifunctional squaramides 8.^23^ The *N*,*N*-bidentate amide unit incorporates an intramolecular hydrogen bond that helps organize the substrate and is important to the observed enantioselectivity (Scheme 4). The proposed stereochemical model accordingly involves recognition of the organized amide substrate by the squaramide catalyst. Subsequent axis-to-center chirality transfer demonstrates the synthetic utility of the allenamide products. The method complements the enyne-to-allenoate chemistry, but its dependence on a deliberately designed amide unit limits direct extrapolation to ordinary enynamides.

**Scheme 4:**
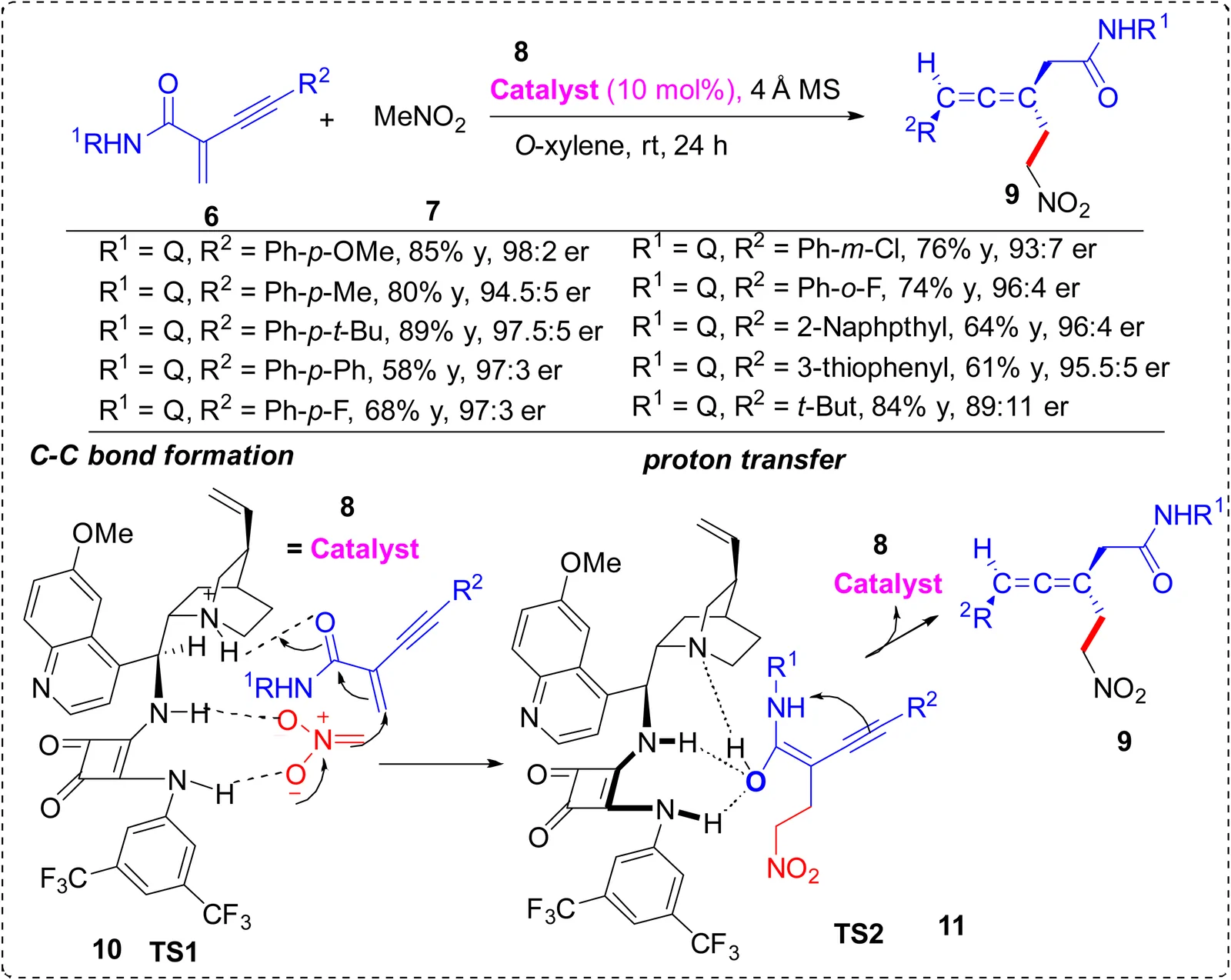
Bifunctional squaramide-catalyzed formation of 2,3-allenamides from hydrogen-bond-stabilized enynamides.^23^

Zhou and co-workers used *bis*-dihydroquinine (DHQ)_2_PYR 14 for γ-addition of pyrazolones to β,γ-alkynyl-α-imino esters 12.^24^ The matched example in Scheme 5 uses 10 mol% catalyst at −20 °C for 12 h and gives 88% isolated yield, >20 : 1 dr and 90% ee. The allene axis and adjacent quaternary stereogenic center are formed together. The general scope procedure allows 12–24 h; it should be distinguished from this specific optimization entry. This approach broadens noncovalent aminocatalysis beyond thiourea- or squaramide-dependent activation.

**Scheme 5:**
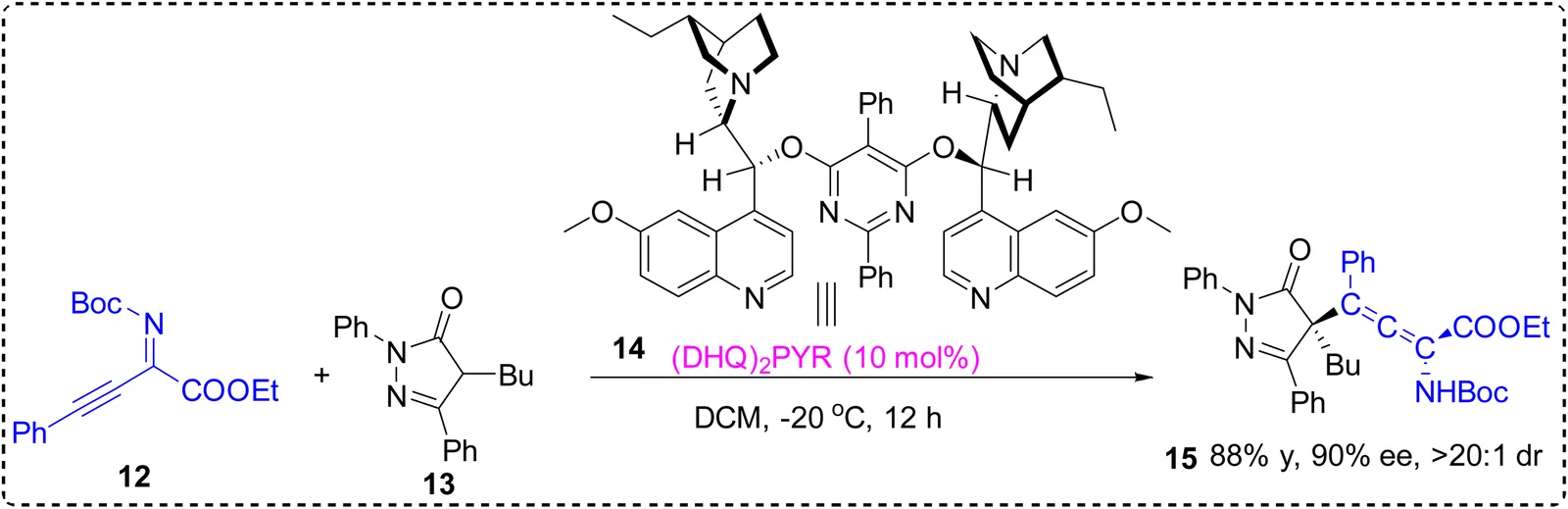
Representative γ-addition of a pyrazolone to an alkynyl imino ester under (DHQ)_2_PYR catalysis.^24^

Zhu and co-workers coupled bismuth(v)-mediated arylation with asymmetric proton transfer under an epi-quinine-derived squaramide 20.^25^Scheme 6 shows a representative α-arylated allene ketone 21 obtained in 81% isolated yield and 90% ee. The triarylbismuth(v) 18 reagent is generated *in situ* before the allenation sequence. Reversible allene/alkyne interconversion and arylation precede enantioselective isomerization. Because the bismuth compound 18 is an arylating reagent, the sequence does not demonstrate catalytic turnover of bismuth.

**Scheme 6:**
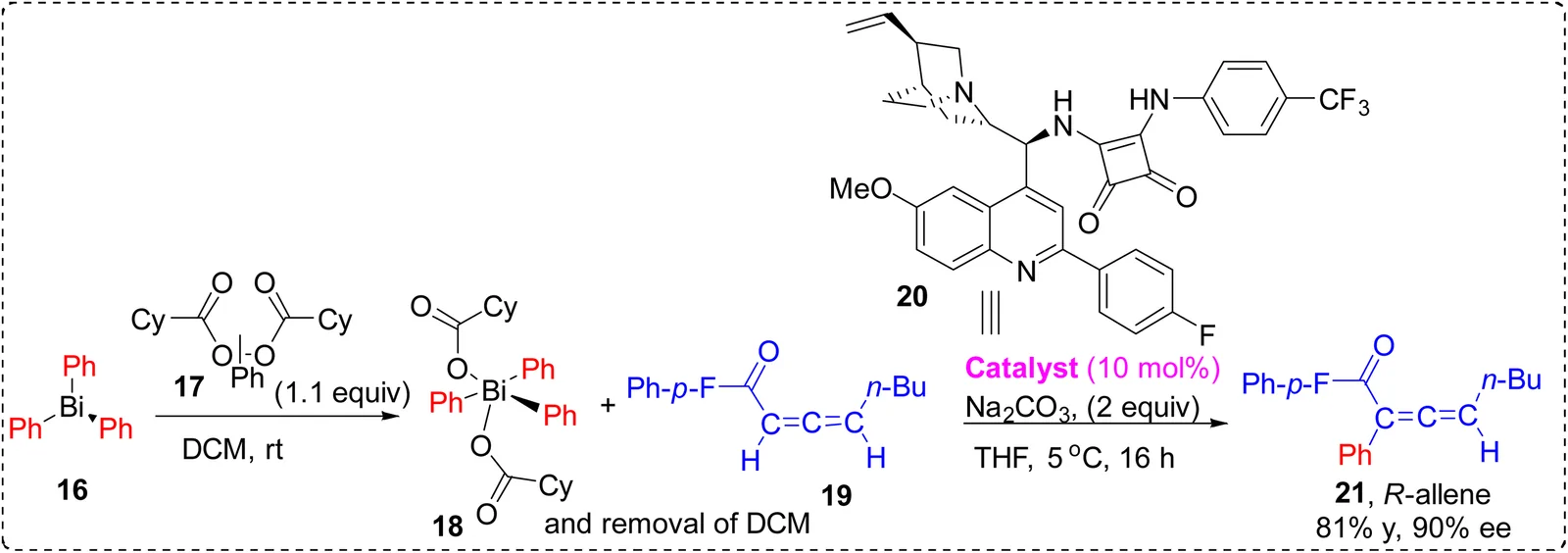
Representative sequential bismuth(v)-mediated α-arylation and squaramide-catalyzed proton transfer, corresponding to source product 3a.^25^

Ji and co-workers reported a Wolff-rearrangement-based reaction of alkynyl diazoketones 22 with anilines 23 in September 2026.^26^ A quinine-derived squaramide 24 favors chiral allenamides 25, whereas a different catalyst/additive combination favors propargylamides. Scheme 7 distinguishes the representative preparative result (75% isolated yield, 91% ee) from the optimization result (80% NMR yield, 91% ee). The aniline is a substrate and the amine-containing squaramide is a catalyst. Direct γ-protonation and a route involving propargylamide formation followed by isomerization remain mechanistic possibilities.

**Scheme 7:**
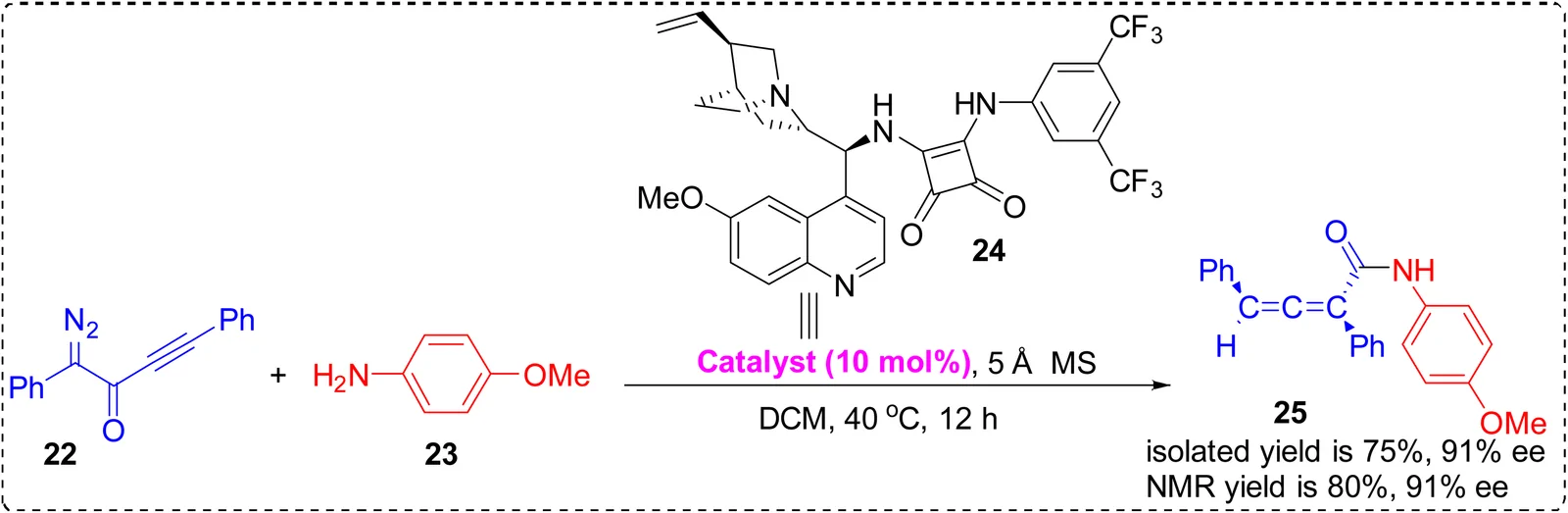
Representative Wolff-rearrangement-based synthesis of an allenamide 25 using *p*-anisidine 23 and an alkynyl diazoketone 22.^26^

### 3.2 Covalent secondary-amine catalysis

Poulsen and co-workers reported the formation of trisubstituted allenes 29 from aldehydes or α,β-unsaturated aldehydes 26 and alkynyl-substituted enones 27 under secondary-amine 28 catalysis.^27^ Saturated aldehydes enter through enamine activation, whereas the enal variants use dienamine activation. The products can contain an allenic axis together with additional stereogenic centers, illustrating simultaneous control of axial and central stereochemistry (Scheme 8). The authors also converted the enamine-derived allene products into chiral furan derivatives, establishing a downstream application of the stereochemically enriched products.

**Scheme 8:**
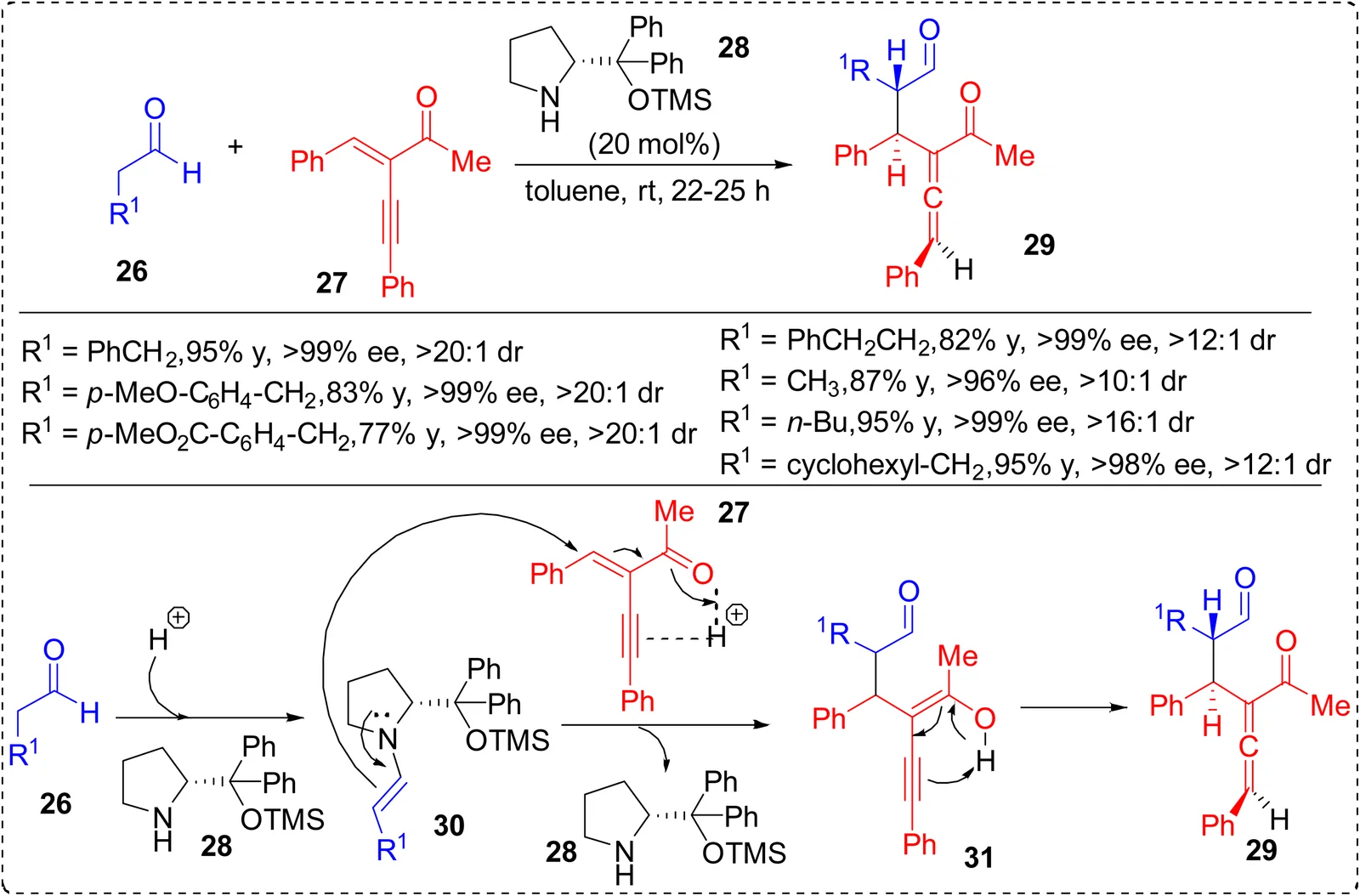
Covalent secondary-amine catalysis for *de novo* formation of chiral trisubstituted allenes 29 from aldehydes or enals and alkynyl-substituted enones.^27^

Reversible condensation of the secondary amine with the aldehyde-derived partner generates the nucleophilic enamine or dienamine, which reacts with the activated alkynyl enone. The proposed bond-forming and proton-transfer sequence produces the allene framework, and hydrolysis releases the carbonyl-containing product and regenerates the amine.^27^ This cycle differs from a propargylamine-mediated covalent relay: the chiral amine is used catalytically rather than being consumed as the stereochemical reagent. Its demonstrated scope nevertheless relies on the complementary reactivity of carbonyl-derived nucleophiles and activated alkynyl enones.

### 3.3 Nucleophilic amine-catalyzed functionalization of pre-existing allene axes

Hu and co-workers described β-isocinchonine-catalyzed asymmetric C(sp^2^)–H allylic alkylation of racemic trisubstituted allenoates 32 with Morita–Baylis–Hillman carbonates 33.^28^ The products 35 retain the cumulated diene and contain both a tetrasubstituted allene axis and an adjacent tertiary stereogenic center (Scheme 9). This is a stereoselective functionalization of a pre-existing allene framework, rather than construction of the cumulene from an alkyne. The substrate and catalyst requirements should be compared with those of direct axis-forming reactions on that basis.

**Scheme 9:**
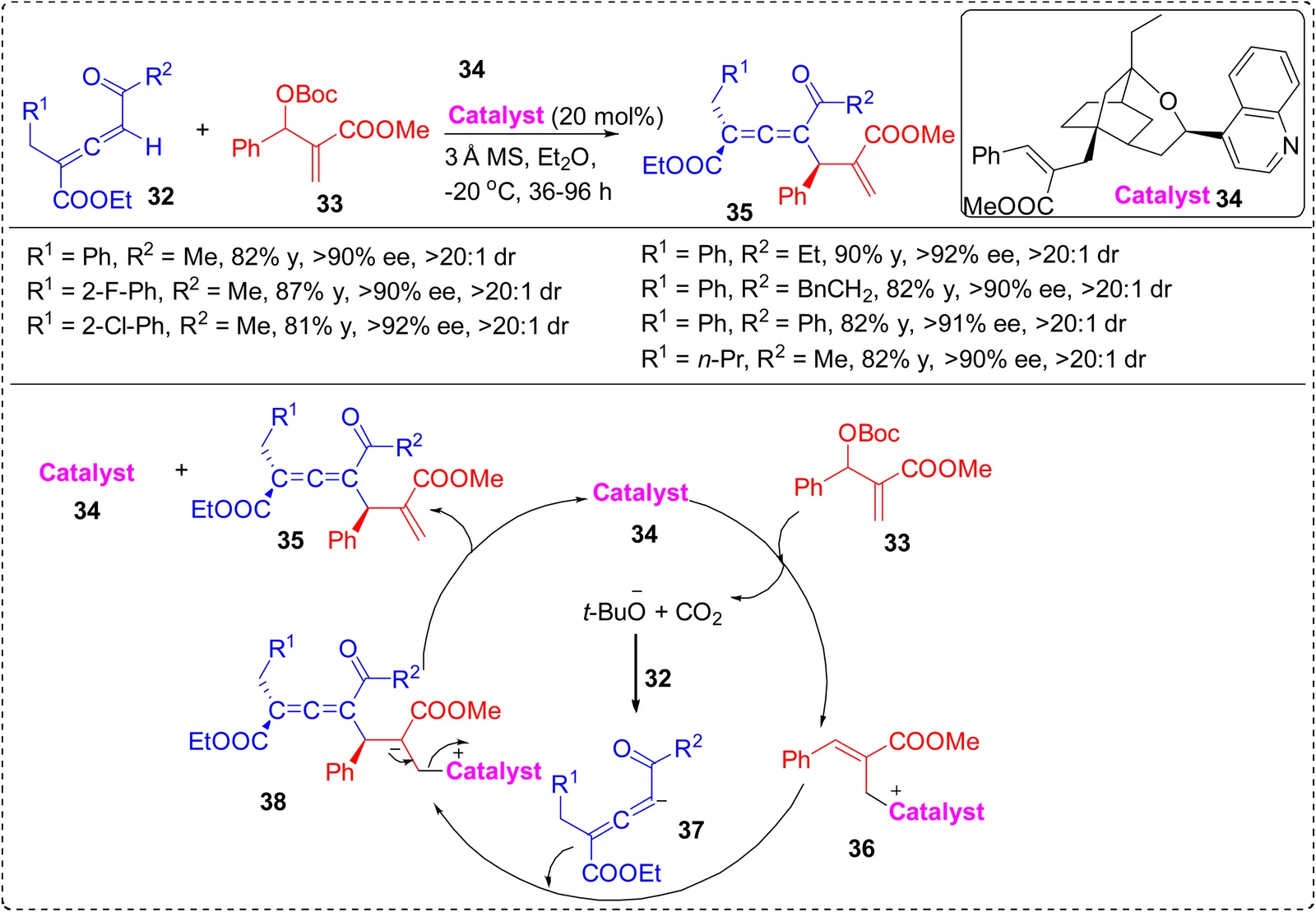
Nucleophilic amine-catalyzed asymmetric allylic alkylation of racemic trisubstituted allenoates 32 with Morita–Baylis–Hillman carbonates 33.^28^

A subsequent computational investigation proposed initial nucleophilic attack of β-isocinchonine on the Morita–Baylis–Hillman carbonate, loss of the carbonate-derived leaving group, stereoselective C–C bond formation, and catalyst regeneration.^29^ The calculations identify C–C bond formation as the stereoselectivity-determining event and implicate C–H⋯O and C–H⋯π interactions in discrimination between competing pathways. These are computational assignments rather than direct observations of transition states. The net conversion of racemic allenoates to stereoselectively functionalized products should not be labeled an ordinary kinetic resolution without evidence for selective consumption of one starting enantiomer and the associated mass balance.

This reaction also clarifies the scope boundary. Chiral-amine-catalyzed annulations that consume the allene and furnish centrally chiral heterocycles are valuable asymmetric reactions, but their isolated products do not retain an allenic axis.^30,31^ They are therefore discussed only where they illuminate activation or chirality-transfer principles relevant to allene synthesis; their heterocycle yields and ee values are not treated as isolated-allene performance data.

### 3.4 Scope and limitations of catalytic chiral-amine control

These studies demonstrate catalytic chiral-amine control through enyne addition, alkynoate isomerization, enamine or dienamine activation, and functionalization of pre-existing allenes.^20–29^ The successful examples require different forms of substrate activation, including ester or ketone groups, an organizing amide unit, or an imino ester. They should consequently be compared by reaction class and substitution pattern rather than ranked by a single maximum ee. The use of catalytic amine loadings is established in these families, but a broadly general catalytic counterpart to stoichiometric alkyne–aldehyde–amine allenation remains a separate objective.

For the modular alkyne–carbonyl platform, catalyst design must integrate selective iminium capture, acetylide addition and rearrangement with regeneration of the active amine. The redox requirement in hydrogen-transfer relays is discussed in Section 2.3. Noncovalent recognition and cooperative metal–amine activation offer design precedents, while the cooperative examples below demonstrate turnover through different reaction networks.^32,33^

## 4 Metal-assisted chemistry and cooperative metal-amine strategies

### 4.1 Cooperative metal-amine catalysis and transient-allene comparisons

You, Shi, Mi, and Luo combined palladium catalysis with chiral primary-amine activation for α-allylic allenylation of β-ketocarbonyls and aldehydes using 1,3-enynes.^33^ The proposed cycle couples a carbonyl-derived enamine with a palladium intermediate, followed by hydrolysis. Changing the amine and ligand configurations provided all four axial/central stereoisomers, although their selectivities differed. This establishes catalytic stereodivergence as an existing capability. Its extension across substrate classes remains important: α-branched aldehydes showed lower diastereoselectivity than many β-ketocarbonyl examples.

Yuan and co-workers reported cooperative gold/quinine-derived squaramide catalysis of reactions between *N*-propargylamides and alkynyl imines to give fully substituted α-amino allenoates.^32^ The general procedure uses 5 mol% gold complex and 10 mol% squaramide in 1,2-dichloroethane at 30 °C overnight. The *N*-propargylamide is a substrate, whereas the quinine-derived amine is a separate catalyst; this differs operationally from a stoichiometric chiral-propargylamine relay.

Control experiments and calculations support an alkylgold-mediated Mannich addition followed by a gold-assisted, stepwise formal [3,3]-σ rearrangement.^32^ Despite the net Michael-type connectivity, the proposed initial bond formation occurs at the imine carbon. Experiments with a preformed alkylideneoxazoline supported cooperation between the gold complex and squaramide rather than a simple relay through that compound. The calculated pathway assists interpretation without establishing every proposed intermediate experimentally.

A related mechanistic comparison is the bifunctional gold ligand developed by Cheng, Wang, Quintanilla, and Zhang.^34^ Its pendant chiral tertiary amine assists proton transfer from a gold-activated alkyne. The proposed chiral allene is then trapped by intramolecular oxygen addition to furnish 2,5-dihydrofurans. The isolated heterocycle stereochemistry and ligand-control experiments support asymmetric induction during the tandem process, but do not constitute a measured yield or ee for an isolated allene. This example illustrates intramolecular metal–amine cooperation while remaining outside the core isolated-allene comparison.

### 4.2 Gold- and silver-assisted rearrangement of chiral propargylamines

Lo, Wong and Che reported the conversion of enantioenriched propargylamines 39 into axially chiral allenes using KAuCl_4_40, with isolated yields up to 93%.^13^ Although the precatalyst contains Au(iii), the proposed rearrangement pathway involves Au(i). The reaction transfers stereochemical information already present in the propargylamine into the allene axis (Scheme 10).

**Scheme 10:**
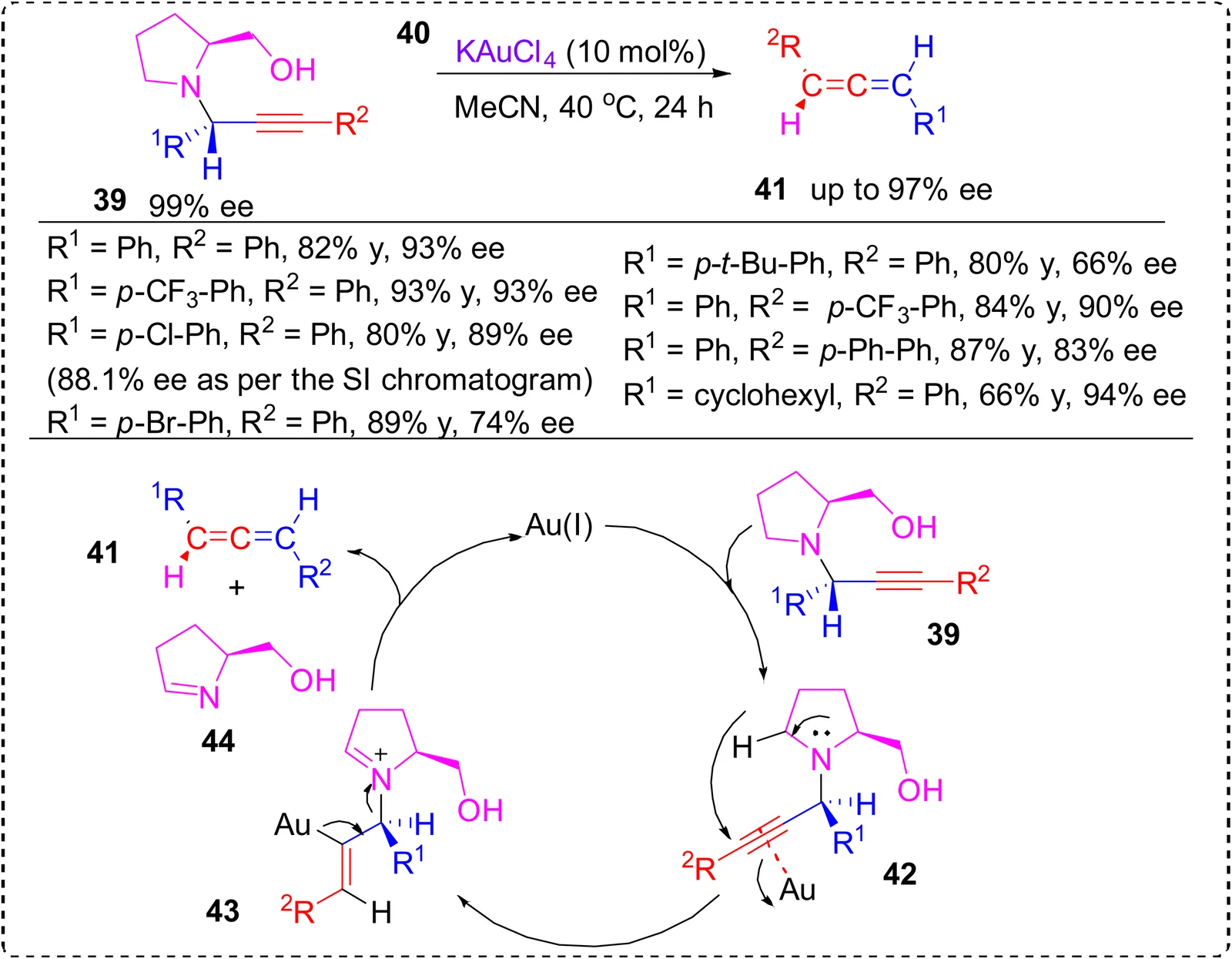
KAuCl_4_-catalyzed conversion of enantioenriched propargylamines 39 into axially chiral allenes 41.^13^

The preformed chiral propargylamine is a covalent stereochemical substrate. For the matched gold example in Table 1, a 99% ee precursor gives allene in 93% ee at 91% conversion; the 82% isolated yield is calculated on converted precursor.^13^ These results support enantioenriched allene formation from an independently prepared precursor. Precursor/product balances and pathway-specific controls would further clarify the extent of stereochemical transfer and the contribution of competing pathways.

**Table 1:** Selected quantitative benchmarks for chiral-amine-enabled allene synthesis. Matched examples, distinct reporting bases and separate scope maxima are identified; the entries are not a direct performance ranking

Platform/example	Amine role and reported conditions	Quantitative outcome and basis	Practical interpretation	Ref.
Nitroalkane addition to an activated enyne: 1a + nitromethane → 3a (source numbering)	Thiourea–tertiary-amine catalyst 4, 20 mol%; CH_2_Cl_2_/MeNO_2_ 4 : 1, 0 °C, 36 h	79% isolated yield; 96% ee; allenoate/alkynoate 12 : 1 before separate isomerization; matched source SI procedure for 3a	Direct catalytic axis construction. A second treatment with catalyst 4 increases allenoate/alkynoate to >25 : 1 without lowering ee (note bb)	22
Hydrogen-bond-organized enynamide allenation: preparative synthesis of 2a	Quinine-derived squaramide 8, 10 mol%; o-xylene/MeNO_2_ 1 : 1, room temperature, 24 h; 4 Å molecular sieves	3.5 mmol enynamide: 1.03 g product, 82% isolated yield (source SI gram-scale procedure). No separate ee is specified for this run	Preparative-scale catalytic synthesis is demonstrated. No ee is inferred from separate small-scale or derivatization experiments	23
Pyrazolone addition to an alkynyl imino ester: 1a + 2a → 3aa	(DHQ)_2_PYR, 10 mol%; CH_2_Cl_2_ (2 mL/0.1 mmol imino ester), −20 °C, 12 h	88% isolated yield, >20 : 1 dr, 90% ee; matched SI optimization entry 26 and product 3aa characterization	Catalytic formation of a tetrasubstituted axis and adjacent quaternary stereocenter	24
Covalent aminocatalysis: aldehyde 1a + alkynyl enone 2a → 4a	Diphenylprolinol trimethylsilyl ether 3a, 20 mol%; benzoic acid, 20 mol%; toluene, room temperature; reported scope protocol 22–25 h	95% reported yield, >20 : 1 dr, >99% ee; matched source Table 2, entry 1. Yield basis retained as reported^a^	Aldehyde added in two portions: 2 equiv. initially and 1 equiv. after 5 h; direct allene product	27
Cooperative gold/squaramide allenation: 1a + 2a → 4a	Me_3_(OMe)tBuXPhosAuNTf_2_, 5 mol%; squaramide 3a, 10 mol%; 1,2-dichloroethane, 30 °C, overnight; 1a/2a = 2 : 1	77% NMR yield, 95% ee; matched optimization Table 1, entry 17	Both catalysts are substoichiometric. NMR yield must be distinguished from isolated yields	32
Cooperative Pd/primary-amine allenylation: product 4e	Pd(PPh_3_)_4_, 5 mol%; L4, 5 mol%; primary-amine catalyst A1, 20 mol%; NaBArF_4_, 6 mol%; EtOAc, 40 °C, 24 h	64% reported yield, 11 : 1 dr, >99% ee; matched source Fig. 3A, product 4e. Yield basis retained as reported^a^	Combines covalent enamine activation with palladium catalysis to form nonadjacent axial and central chirality	33
Gold-catalyzed rearrangement of enantioenriched propargylamine 1a	Covalently bound chiral prolinol unit; KAuCl_4_, 10 mol%; CH_3_CN, 40 °C, 24 h	Allene 2a: 82% isolated yield based on 91% conversion; 93% ee from 99% ee precursor; matched SI Table S1, entry 1	Metal catalysis acts on a preformed chiral substrate. Conversion-based yield is not directly comparable with charged-substrate yields	13
Preparative α-allenol: TBS-protected propargyl alcohol + cyclohexane-carboxaldehyde	(*S*)-α,α-Diphenylprolinol, 100 mmol; ZnBr_2_, 76.6 mmol; toluene, 130 °C oil bath, 11 h; then TBAF·3H_2_O, 101 mmol, THF, room temperature, 2 h	9.42 g, 62% combined isolated yield over two steps, 99% ee; yield based on 100 mmol prolinol	Independently checked preparative procedure. Alkyne and aldehyde amounts are 110 and 150 mmol, respectively	40
Matched mediator comparison: pent-4-yn-1-ol 1c + n-C_11_H_23_CHO 2a	Alkynol/aldehyde/amine = 1.5 : 1.5 : 1; CuBr_2_, 20 mol% relative to amine; 1,4-dioxane, 130 °C, 12 h	Diphenylprolinol: 56% isolated yield, 85% ee. Dimethylprolinol: 53% isolated yield, 95% ee. Both from the same substrate pair (source Table 1, entry 3)	The higher ee with dimethylprolinol is not accompanied by a higher yield for this matched pair. Yields are based on the limiting amine	38
*N*-Methylcamphanyl-piperazine relay: phenylacetylene + benzaldehyde	Stoichiometric covalent mediator; ZnCl_2_-promoted propargylamine formation followed by ZnI_2_-mediated allenation in toluene	Propargylamine: 90% reported isolated yield; rearrangement: 52% reported isolated yield, 96% ee, for the same diphenylallene sequence. The step and isolated yields are not an overall yield^a^	The 52% value describes the rearrangement step, not the overall yield from alkyne and aldehyde. Regioisomeric templates provide stereodivergent access	43
Chiral-piperazine ZnCl_2_/ZnBr_2_ relay: scope summary	Stoichiometric covalent amine relay; ZnCl_2_ for precursor formation and ZnBr_2_ for rearrangement; reported allenation time 1–2 h	Propargylamines: 67–95% isolated yield, up to 99 : 1 dr. Allenes: up to 89% isolated yield and up to 99% ee (separate scope maxima)	79–86% isolated piperazine recovery follows NaBH_4_ reduction of imine-derived material. Recovery is measured after allenation and reduction; repeated reuse and *in situ* amine turnover require separate evaluation	17
2-Dialkylamino-methylpyrrolidine relay: scope summary	Stoichiometric covalent mediator; CuBr-promoted precursor formation at 25 °C; CuI-mediated rearrangement in dioxane at 100 °C	Propargylamines: up to 96% yield and 99 : 1 dr. Allenes: up to 81% yield and 99% ee (separate scope maxima)	A two-stage sequence separates stereocenter formation from axial-chirality transfer; the quoted maxima are not a matched substrate pair	46
Cyclic chiral-amine route to trisubstituted allenes: scope summary	Stoichiometric 2-benzylmorpholine or *N*-methylcamphanyl-piperazine relay; CuCl, 110–120 °C, then ZnBr_2_, 120 °C	Diastereomerically pure chiral propargylamines: 23–89% yield. Subsequent allenation: 71–89% yield, up to 99% ee	Reported allenation yields refer to isolated propargylamines and should not be read as overall yields for the two-stage sequence	45
*N*-Benzylated decahydro-quinoxaline relay: 1-decyne + benzaldehyde	Chiral amine, 1.0 equiv.; ZnI_2_, 0.5 equiv.; toluene. 120 °C, 4 h; aldehyde addition at 120 °C (author clarification; note cc)	Matched product 8a: 70% isolated yield, 90% ee. A separately reported aromatic-alkyne example gives 25% yield, 96% ee	Direct amine recovery was not achieved under the one-pot conditions examined. Separate isolated-propargylamine rearrangement/NaBH_4_ treatment gave 75% regenerated amine; performance over successive reuse cycles requires separate evaluation^d^	19
Bi(v)-mediated arylation/asymmetric proton transfer: source product 3a	Epi-quinine squaramide, 10 mol%; Bi(v) arylating reagent generated from BiPh_3_ (0.10 mmol); ketone, 0.15 mmol; Na_2_CO_3_, 2 equiv.; THF, 5 °C, 16 h	81% isolated yield; 90% ee; matched source Fig. 3, product 3a	Bi(v) is a reagent; only the squaramide is organocatalytic. Proposed isomerization/arylation/proton-transfer sequence; see Scheme 6	25
Wolff rearrangement/allenamide formation: source product 3a	Quinine squaramide, 10 mol%; diazoketone/aniline 2 : 1; 5 Å MS; CH_2_Cl_2_, 40 °C; preparative procedure ends at TLC completion	75% isolated yield, 91% ee (SI p. S8); separately, from the optimization results of 80% NMR yield, 91% ee (SI Table S1, entry 11)	The preparative and optimization yields are different experiments. 12 h time is assigned to the general procedure; see Scheme 7	26

a Compound and catalyst labels in this table identify the cited primary-source examples except compound/catalyst numbers 4 and 8. Paired yield/ee values belong to the same stated experiment; scope maxima may describe different substrates and are not matched. Loadings retain the source denominator. Unless explicitly described as isolated, NMR, conversion-based or combined multistep yield, a value is retained only as a reported yield and is not assumed to have a particular basis. No unlisted scale, recovery yield, catalyst reuse or reaction time is inferred.

b For the nitroallenoate example, the SI reports 79% yield and 96% ee with an initial 12 : 1 allenoate/alkynoate ratio. A separate treatment with 20 mol% 4 in CH_2_Cl_2_ at room temperature for 36 h gives >25 : 1 without loss of ee. Thus the 36 h initial reaction should not be presented as the complete time for the additional purification-by-isomerization sequence.

c The correct reaction conditions in ref. 19 are 120 °C for 4 h, with aldehyde addition also performed at 120 °C, as confirmed by the corresponding author. The 12 h stated in the general procedure and 130 °C stated in the optimization-table footnote are typographical errors. Table 1 uses these author-clarified conditions.

d Ref. 19: the initial chiral amine is 1.0 equiv. in the direct one-pot operation. The reported 75% recovery instead follows rearrangement of independently isolated propargylamine and NaBH_4_/MeOH reduction of the imine-containing mixture. Direct amine recovery from the one-pot mixture was not achieved under the conditions examined. No turnover number or series of reuse cycles is inferred from this recovery yield.

**Table 2:** Scope of selected earlier reviews and the complementary focus of this review

Review	Primary scope	Organizing focus	Boundary relative to this review
Chu *et al.*, 2017 (ref. 6*b*)	Catalytic asymmetric synthesis of allenes	Catalytic routes across catalyst families	A broad catalytic perspective; stoichiometric amine relays are not its organizing subject
Wang *et al.*, 2022 (ref. 9)	Organocatalytic synthesis of axially chiral allenes	Organocatalytic activation and stereocontrol	A catalyst-centered account; the present comparison also follows stoichiometric mediator fate
Woldegiorgis *et al.*, 2024 (ref. 10)	Chiral allenes and styrenes through phosphoric acid catalysis	Chiral Brønsted acid activation	A different catalyst class; useful as an external stereocontrol benchmark
Li *et al.*, 2024 (ref. 11)	Asymmetric 1,4-difunctionalization of 1,3-enynes	Radical and non-radical strategies	Transformation-defined coverage rather than an assessment organized by amine function
Present review	Catalytic chiral-amine control and stoichiometric covalent chirality transfer	Amine role, stereochemical evidence, recovery/reuse and quantitative reporting	A focused cross-strategy framework; not a comprehensive survey of all asymmetric allene chemistry

The silver(i)-mediated protocol of Lo, Zhou, Wong and Che provides thermal and microwave-assisted alternatives, generally giving 96–99% ee, with one reported example at 91% ee.^14^ The reported product-control experiments found no detectable racemization under the conditions tested. This is substrate- and condition-specific evidence for product stability; it does not independently exclude precursor epimerization, unequal precursor conversion or selective decomposition. Both protocols begin with a covalently bound chiral propargylamine 39 (Scheme 11).

**Scheme 11:**
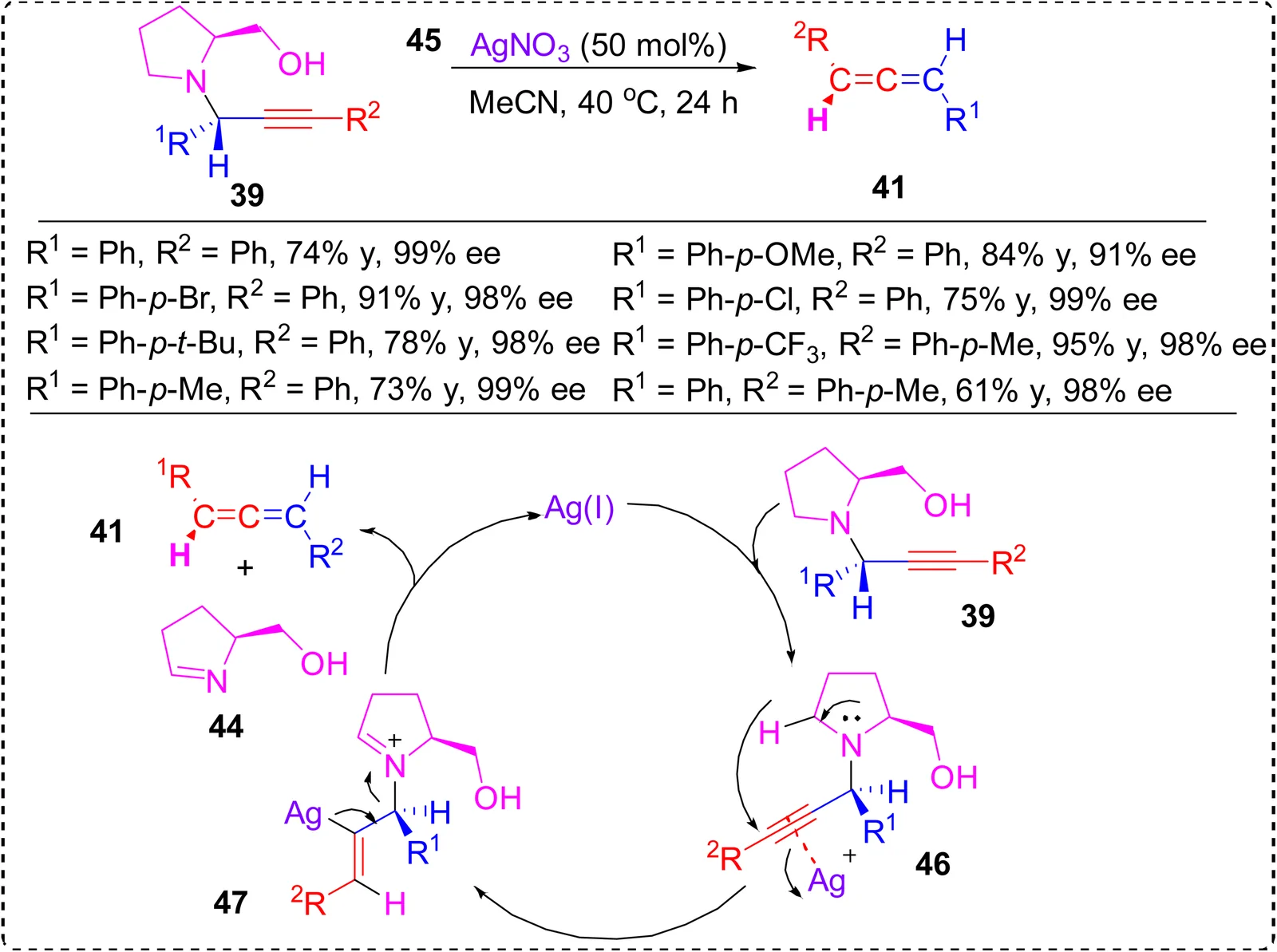
Silver(i)-mediated conversion of optically active propargylamines 39 into axially chiral allenes 41.^14^

### 4.3 Silver-mediated vinylallene synthesis using a prolinol auxiliary

Lo, Chan, Zhou, Toy and Che subsequently employed (*S*)-prolinol as a chiral auxiliary for AgNO_3_-mediated preparation of axially chiral vinylallenes under microwave irradiation.^15^ Selected products exceeded 99% ee. Subsequent (hetero–Diels–Alder reactions with azodicarboxylates or maleimides gave products with up to 99% ee, connecting auxiliary-controlled allene synthesis with axial-to-central chirality transfer. The stoichiometric prolinol auxiliary remains distinct from catalytic amine turnover (Scheme 12).

**Scheme 12:**
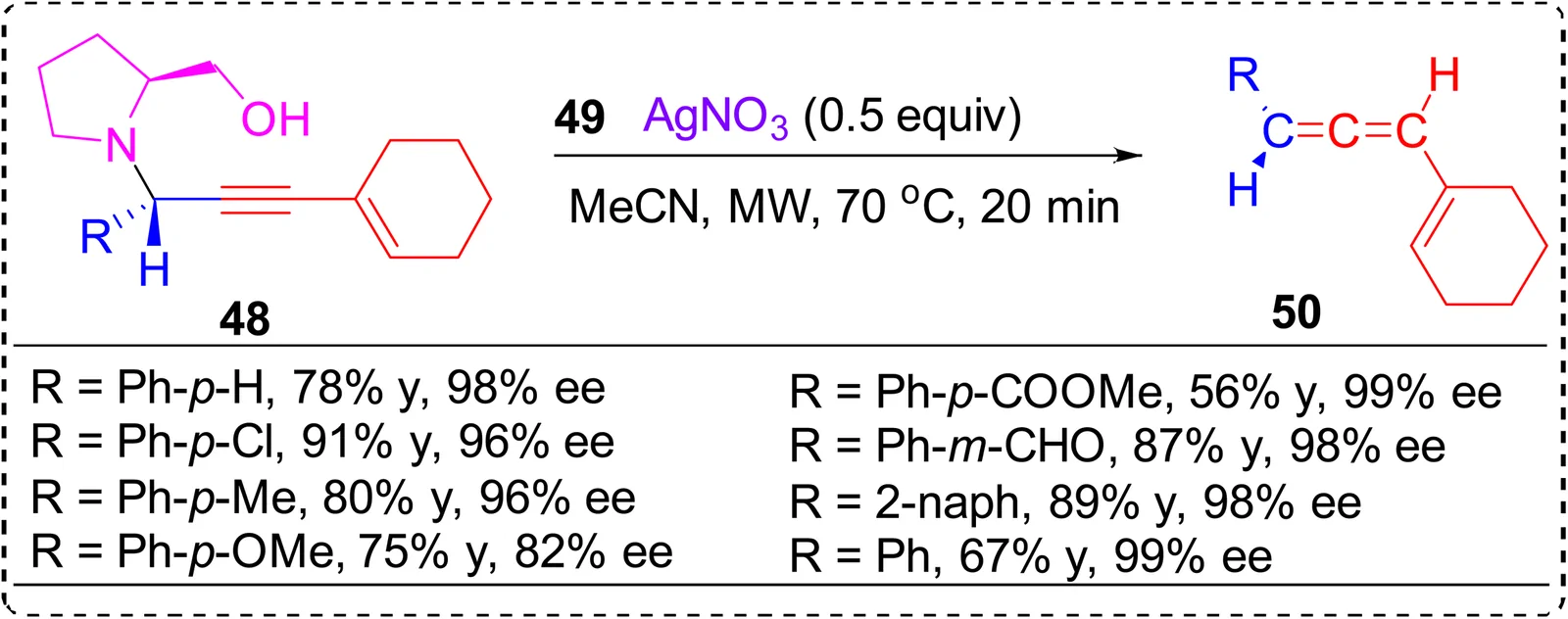
Silver-mediated vinylallene 50 synthesis using a prolinol auxiliary 48.^15^

### 4.4 Copper- and zinc-assisted allenation

The copper/zinc protocols illustrate two different sources of stereocontrol. In the 2012 study by Ye and co-workers, achiral pyrrolidine 53 was combined with a chiral ligand 54, CuBr and ZnI_2_ to convert propargylic alcohols 51 and aldehydes 52 into axially chiral allenols 55 with up to 97% ee.^35^ Coordination of the substrate alcohol contributed to the observed selectivity. This ligand-controlled reaction provides a useful benchmark for the chiral-amine-mediated routes (Scheme 13).

**Scheme 13:**
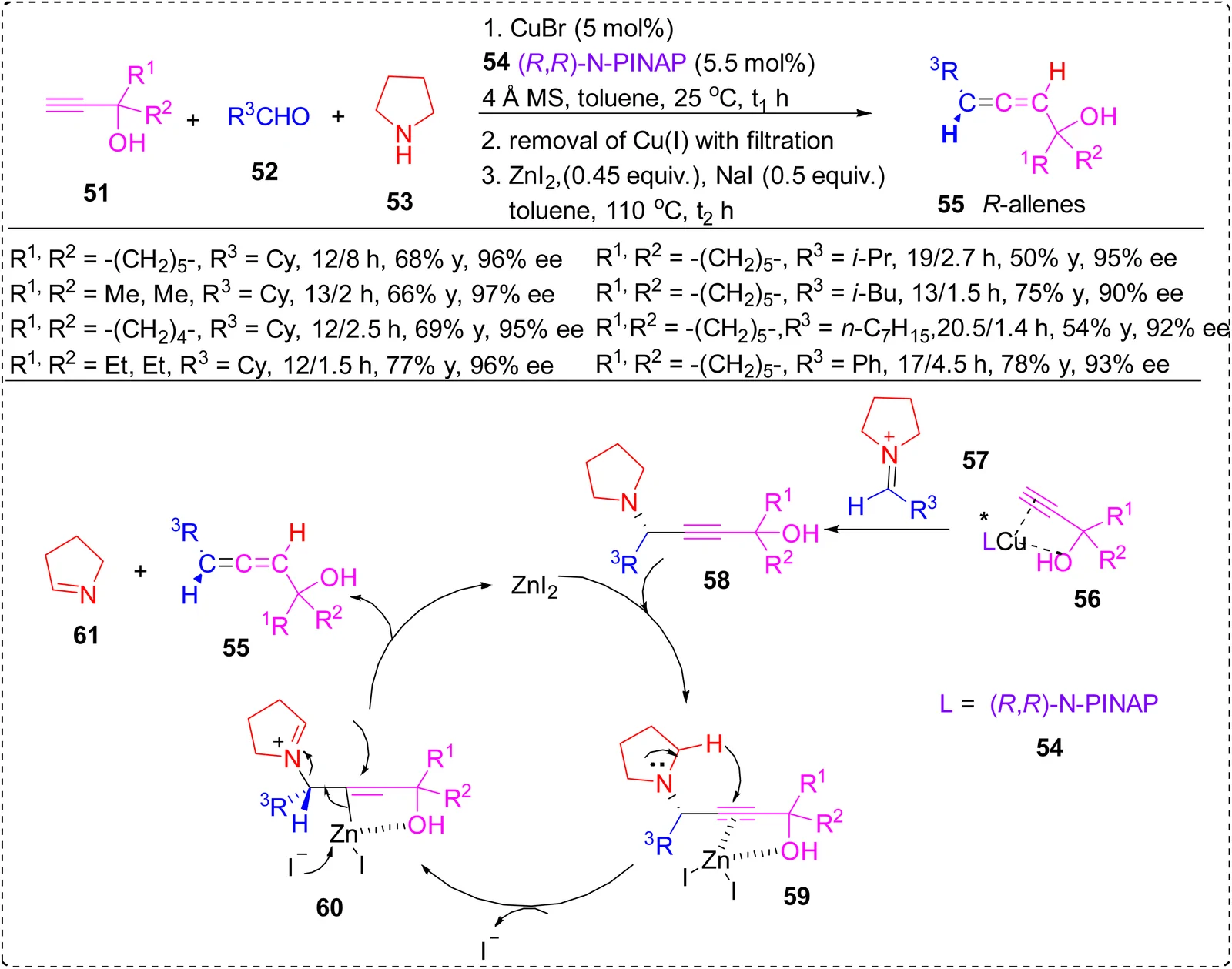
Chiral-ligand-controlled copper/zinc synthesis of axially chiral allenols 55 using achiral pyrrolidine 53.^35^

Lü and co-workers used (*S*)-α,α-diphenylprolinol as the stoichiometric source of chirality in a one-pot combination of terminal alkynes and aldehydes.^36^ Control experiments assigned CuBr a role in efficient propargylamine formation and showed that the combination of CuBr and ZnBr_2_ was important for the amine-to-allene transformation. Copper therefore contributes to both precursor formation and the subsequent rearrangement (Scheme 14).

**Scheme 14:**
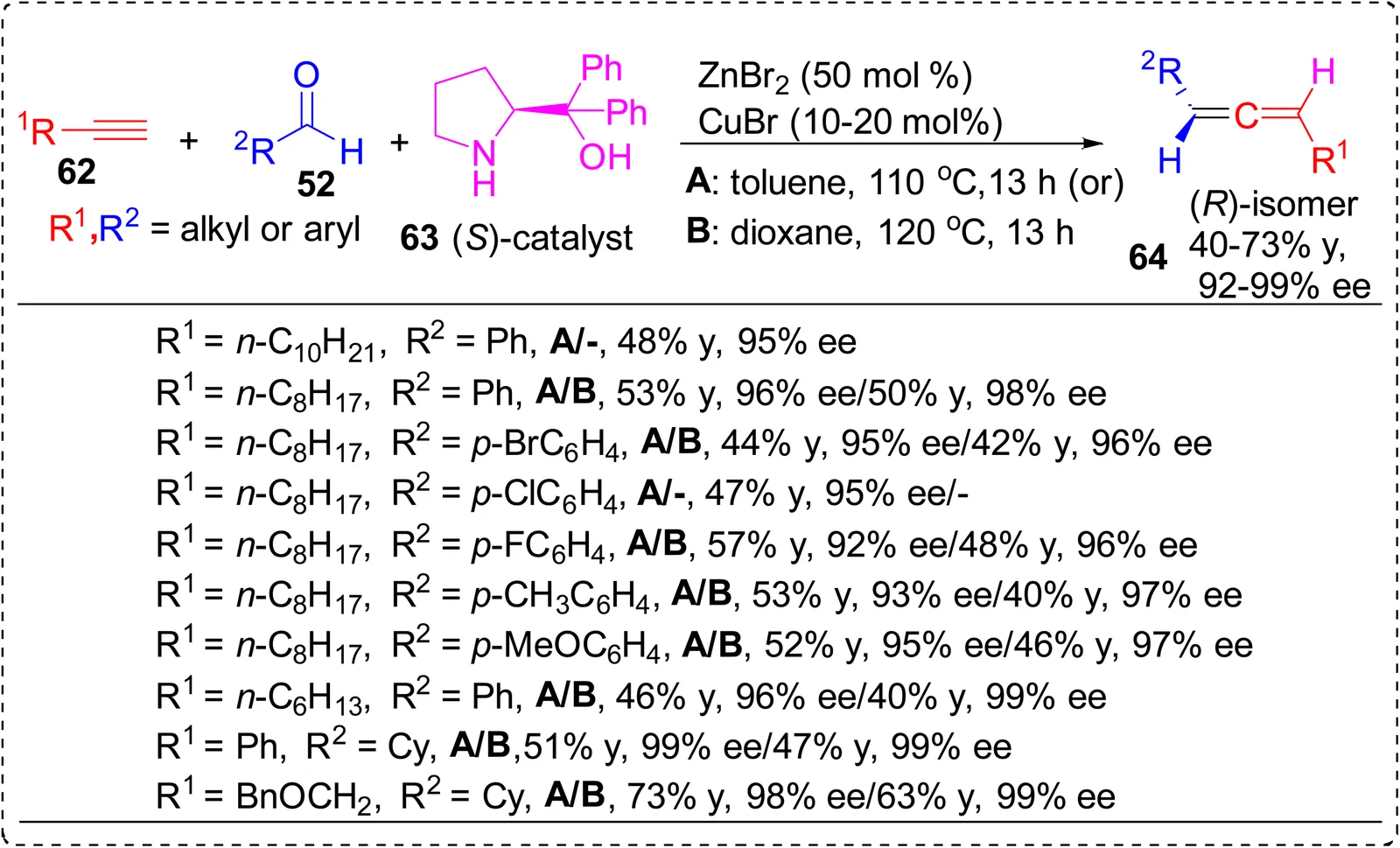
CuBr/ZnBr_2_-assisted one-pot allenation using stoichiometric (*S*)-α,α-diphenylprolinol 63 as a chiral mediator.^36^

The bimetallic protocol reduces intermediate-isolation operations while accommodating the reported alkyne 62 and aldehyde 52 scope.^36^ Its stoichiometric diphenylprolinol 63 requirement places it within covalent mediation under the definitions in Section 2.2.

### 4.5 Chiral allenols and the effect of prolinol structure

Huang and co-workers established a CuBr_2_-catalyzed direct synthesis of optically active allenols 66 from terminal alkynols 65, aldehydes 52 and either enantiomer of α,α-diphenylprolinol 63.^37^ The stoichiometric chiral amino alcohol controls the product configuration. This method established the diphenylprolinol platform subsequently used to investigate the effect of prolinol substitution on stereoselectivity.

Ma, Duan, Fu, Huang and Ma subsequently compared α,α-dimethylprolinol with α,α-diphenylprolinol in CuBr_2_-catalyzed allenylation of terminal alkynols.^38^ Dimethylprolinol improved the ee for the directly compared substrates, showing that increased mediator steric bulk does not necessarily improve stereocontrol. Both aliphatic and aromatic aldehydes were examined, with modified conditions required for the aromatic series. Variation of the alkynol tether extended the product family beyond α-allenols to more remote hydroxy-substituted allenes (Scheme 15).

**Scheme 15:**
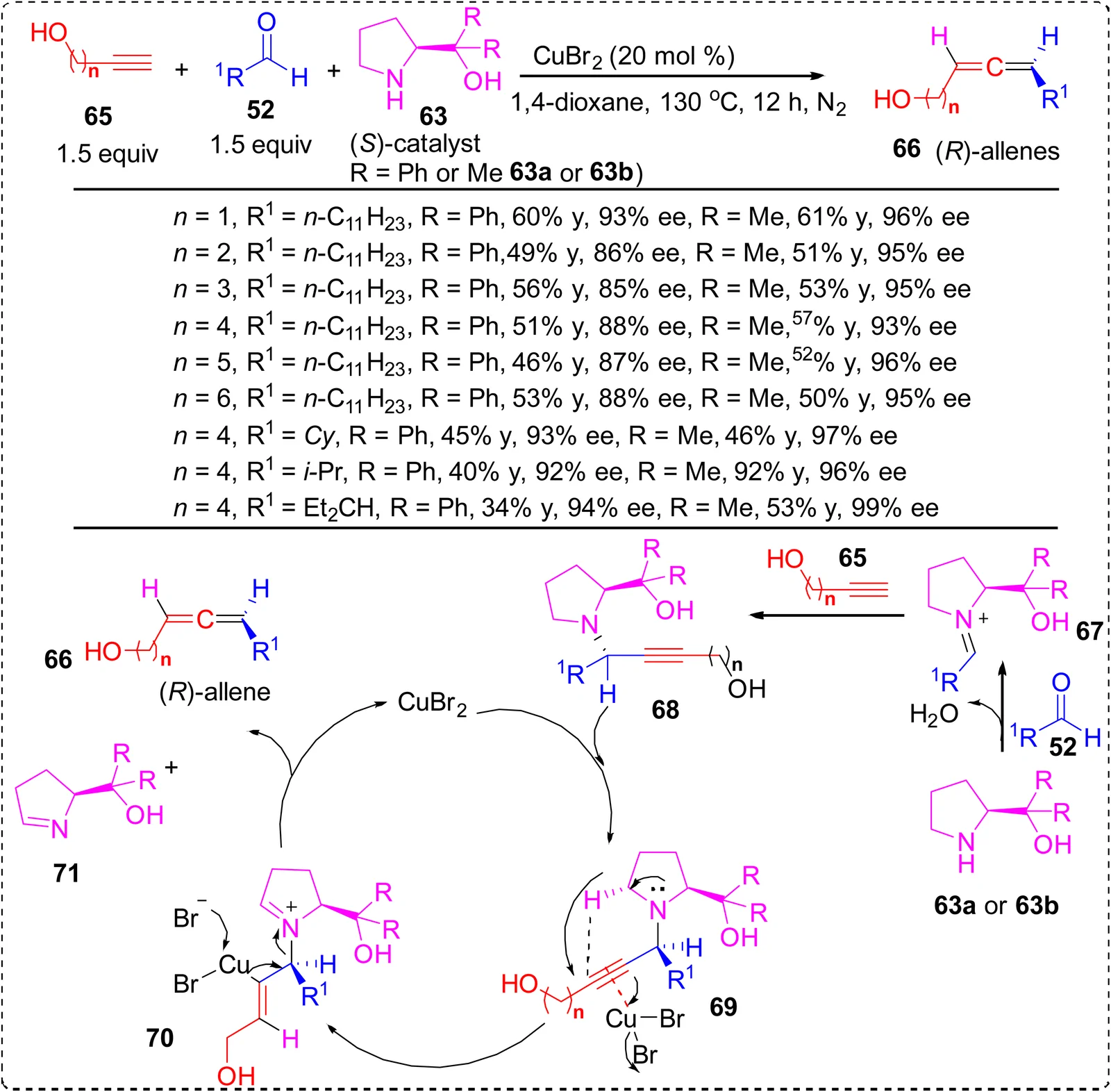
Matched CuBr_2_-catalyzed allenylation examples comparing (*S*)-α,α-diphenylprolinol 63b and (*S*)-α,α-dimethylprolinol 63a.^38^

Under representative aliphatic-aldehyde conditions, CuBr_2_ was used at 20 mol% with stoichiometric prolinol; aromatic aldehydes required up to 50 mol% CuBr_2_.^38^ A 10 mmol preparation afforded an allenol 66 in 55% yield and 97% ee. Subsequent transformations included a four-step synthesis of phlomic acid 72, illustrating preparative utility without establishing catalytic turnover of the amine (Scheme 16).

**Scheme 16:**
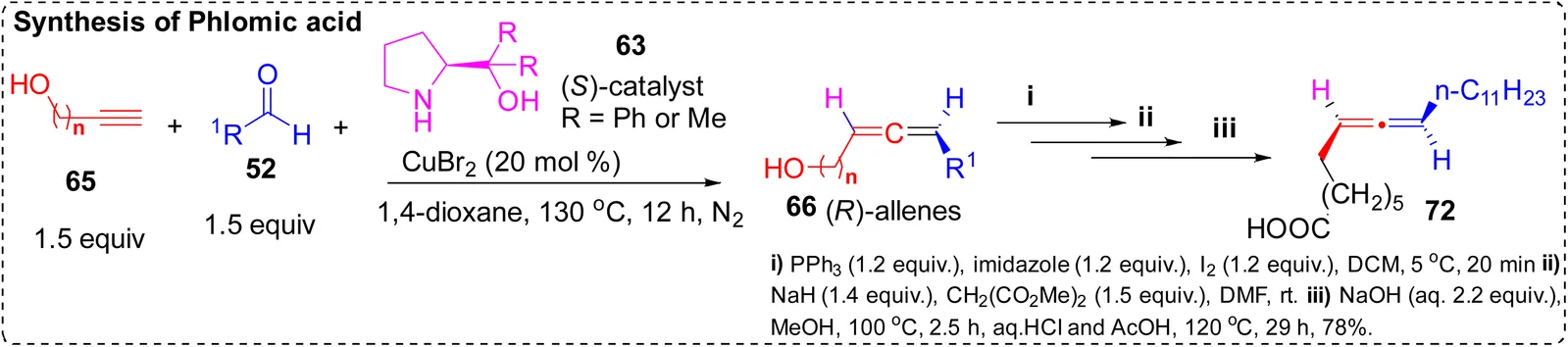
Preparative allenol synthesis and its application to phlomic acid 72.^38^

The protected-alkynol strategy developed by Ye, Fan and Ma provides a complementary solution for α-allenols 76.^39^ TBS-protected propargylic alcohols 73, aldehydes 74 and either enantiomer of α,α-diphenylprolinol 63b furnished allenols after desilylation. For secondary α-allenols, selecting the configurations of both the starting propargylic alcohol and the amine gave access to all four combinations of central and axial stereochemistry. The subsequent checked organic syntheses preparation established a 100 mmol procedure for a representative primary α-allenol: allenation and deprotection gave 62% overall yield and 99% ee, with 65% yield in a second run (Scheme 17).^40^

**Scheme 17:**
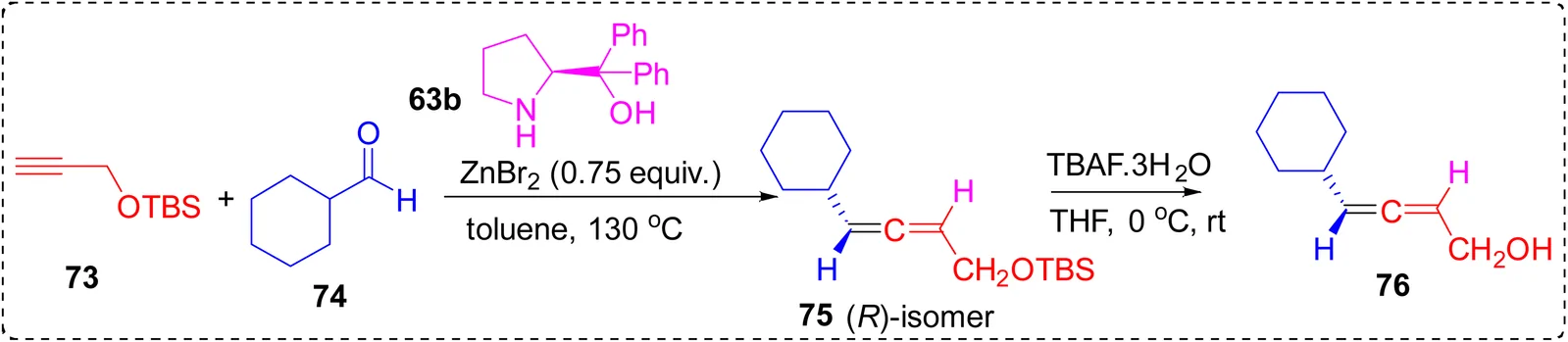
Zinc-bromide-mediated preparation of a primary α-allenol 76 from a TBS-protected propargylic alcohol 73, followed by desilylation.^39,40^

Periasamy and co-workers reported a zinc-halide-promoted one-pot combination of terminal alkynes 62, aldehydes 52 and chiral secondary amines 77 in toluene at 120 °C, giving allenes 80 in reported yields up to 77% and enantioselectivities up to 99% ee.^16^ Diastereoselective propargylamine formation precedes central-to-axial chirality transfer within the zinc-promoted sequence. Reductive regeneration of the amine-derived fragment is a separate operation from allenation and is not evidence of amine turnover during the reaction (Scheme 18).

**Scheme 18:**
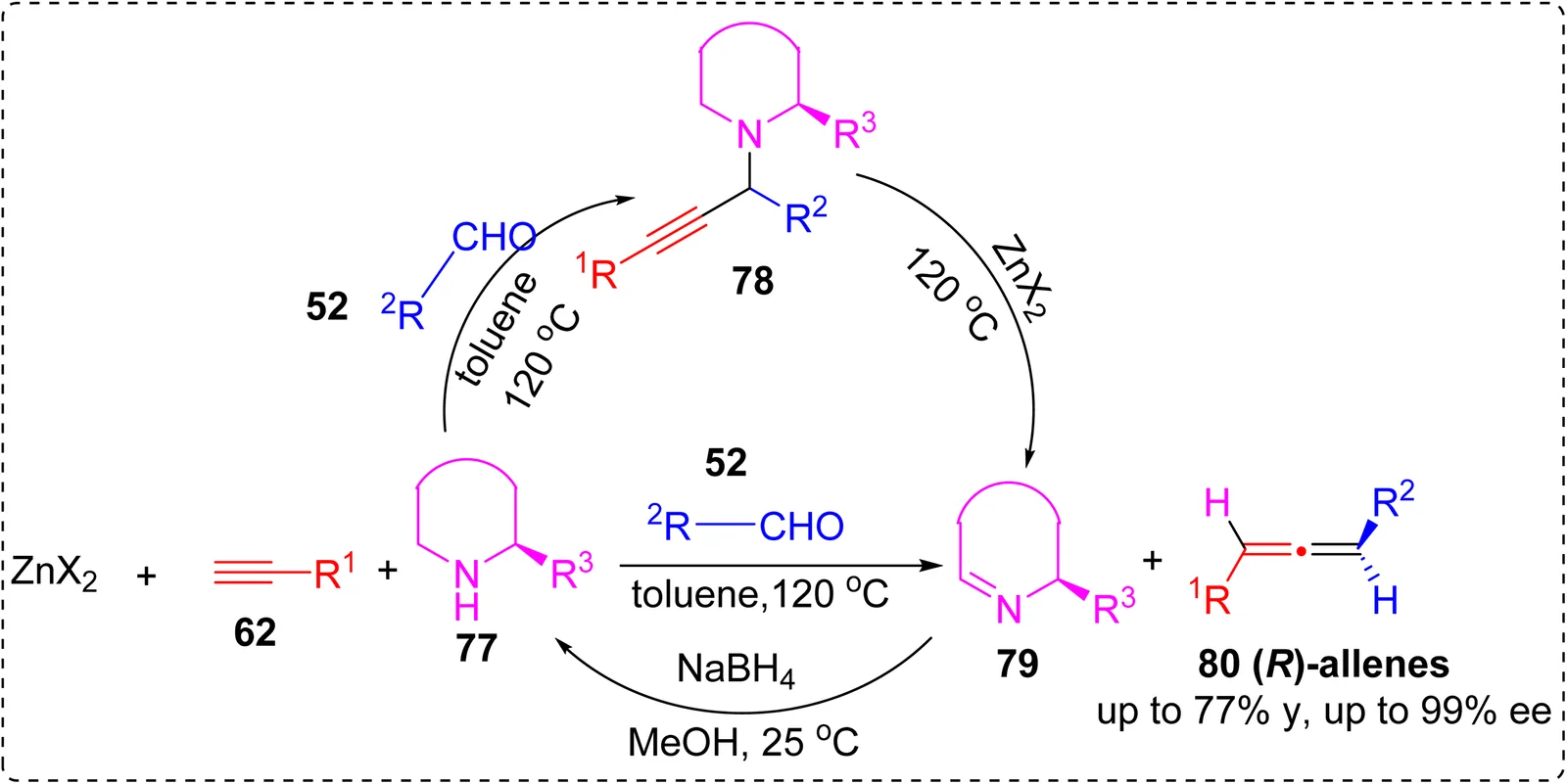
Zinc-halide-promoted one-pot allene 80 synthesis through propargylamine 78 formation and chirality transfer; amine regeneration is a separate reductive operation.^16^

The aryl-substituted zinc/prolinol reaction family was subsequently examined by Ye, Lü, Fan and Ma.^41^ In the Organic Syntheses account, Ye and Ma reported that the yield and ee of an earlier aryl-allene procedure were not reproduced in their experiments and noted differences in calibrated specific rotations.^40^ These observations highlight the importance of comparing matched substrates under clearly specified reaction and analytical conditions.

These examples distinguish chiral-ligand control, stoichiometric prolinol mediation and substrate–mediator combinations that establish both central and axial stereochemistry.^16,35,36,38–40^ Their relative value depends on the required substitution pattern, number of operations and complete reagent balance.

### 4.6 Cyclohexenylallenes and transfer of axial information

Satyanarayana, Mohan and Periasamy reported a copper(i)-catalyzed synthesis of chiral cyclohexenylallenes 50 from 1-enynes 62, aldehydes 52 and chiral secondary amines 28.^42^ Across the substrate scope, yields reached 79% and enantioselectivities reached 99% ee. The enyne substrate gives a conjugated allene architecture suitable for subsequent cycloaddition (Scheme 19).

**Scheme 19:**
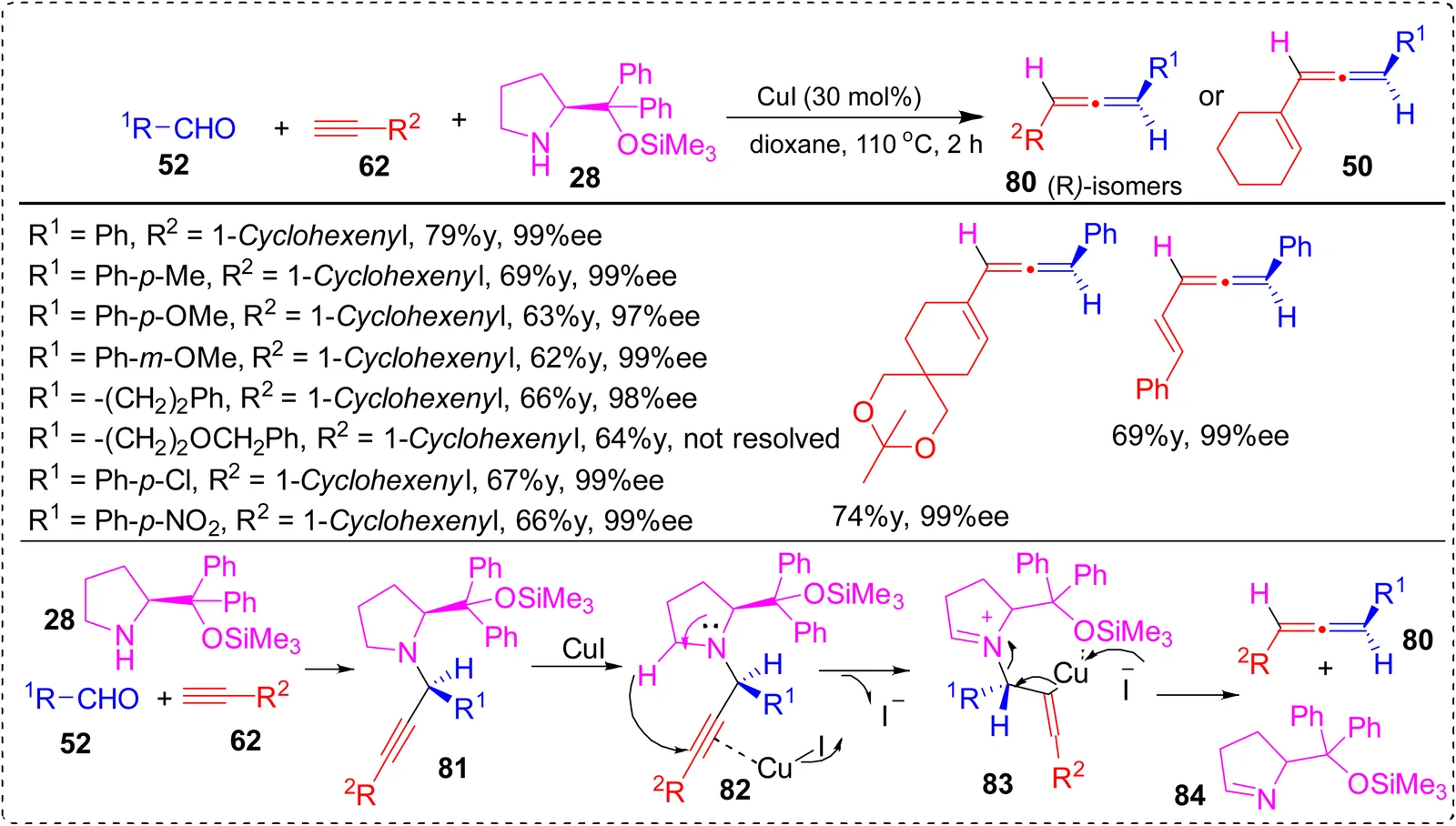
Copper(i)-catalyzed synthesis of chiral cyclohexenylallenes 50 using chiral secondary-amine mediators 28.^42^

The same study demonstrated ytterbium(iii)-catalyzed [4+2] cycloaddition with axial-to-central chirality transfer.^42^ The conjugated allene architecture is introduced in Scheme 19; its subsequent cycloaddition is discussed with the synthetic applications in Section 7.1.

## 5 Stoichiometric chiral-amine-mediated synthesis

### 5.1 Camphanyl piperazine platforms

Periasamy, Reddy and Sanjeevakumar showed that regioisomeric *N*-methylcamphanyl piperazine 85 templates could provide opposite enantiomers of 1,3-disubstituted allenes 80.^43^ Diastereoselective propargylamine formation and zinc-promoted chirality transfer furnished products in reported yields of 38–75% and with 79–99% ee. In the zinc/piperazine study, the configuration of the newly formed propargylic centre in propargylamine 18aej (source numbering; CCDC 1008592) was established by single-crystal X-ray analysis. The allene configurations were assigned using the Lowe–Brewster rule and comparison of optical rotations with literature values.^17^ The ability to select the allene enantiomer through the piperazine regioisomer is a useful feature of this covalent-relay platform (Scheme 20).

**Scheme 20:**
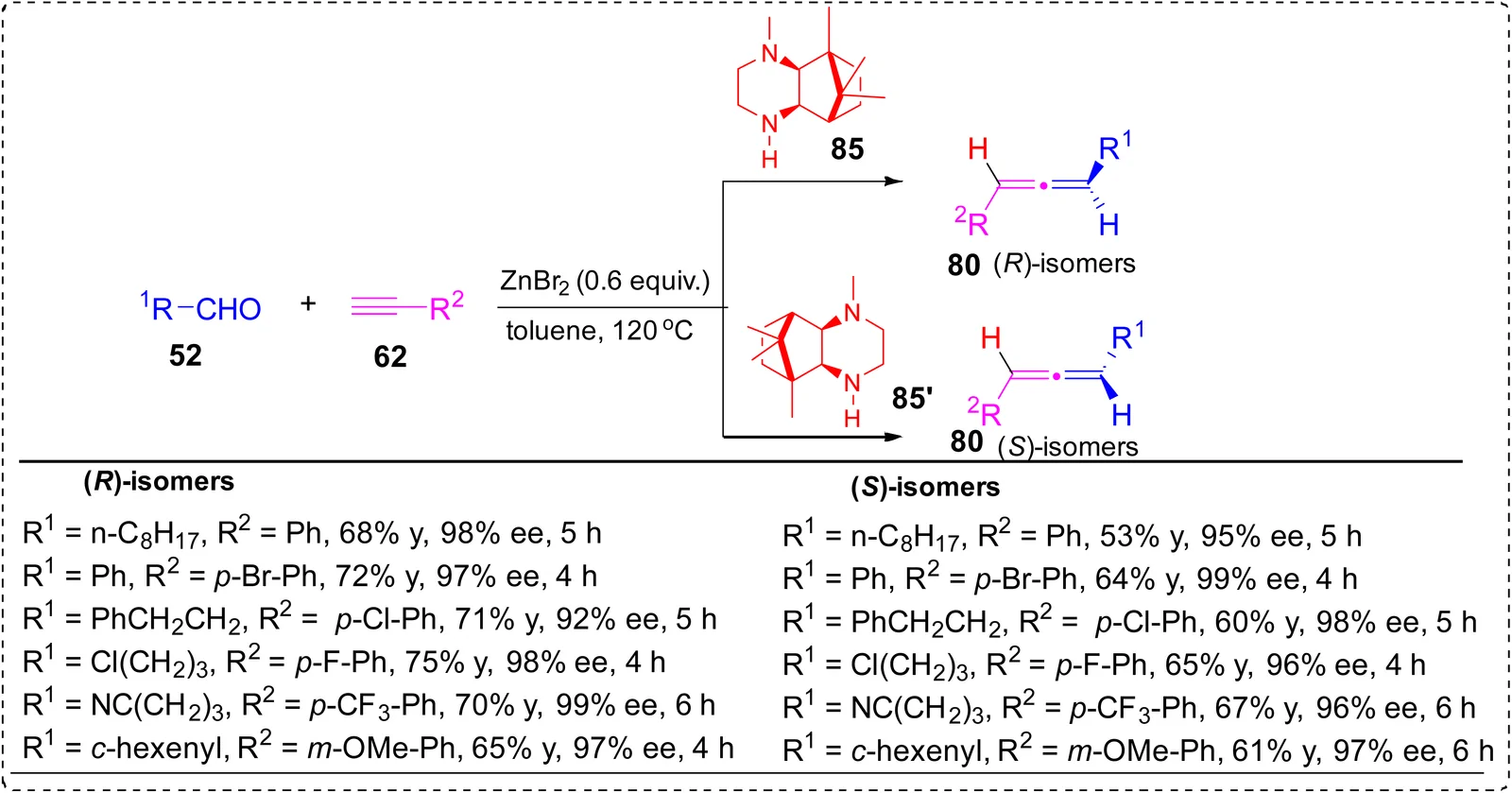
Regioisomeric *N*-methylcamphanyl piperazine 85 templates providing access to opposite allene enantiomers 80.^43^

In the chiral-piperazine study, ZnCl_2_-promoted formation of propargylamines 89–91 afforded 67–95% yields and dr values up to 99 : 1. Subsequent ZnBr_2_-promoted rearrangement required 1–2 h, with separate scope maxima of 89% allene yield and 99% ee.^17^ The piperazine was used stoichiometrically (piperazine/alkyne/aldehyde = 1.0 : 1.1 : 1.0), and *in situ* NaBH_4_ reduction of the imine byproducts after allenation afforded 79–86% isolated piperazine recovery, relative to the initial piperazine amount used (Scheme 21). This experiment demonstrates regeneration after reduction and work-up; successive reuse cycles and catalytic amine turnover were not reported. A specific product-stability limitation was the cyclodimerization of 1,3-diphenylallene 7cea (source numbering) under neat conditions at 25 °C within 24 h, supporting preparation of sensitive allenes from isolated propargylamines when required.^17^ Functionalized-allene applications are presented separately in Scheme 22.^44^

**Scheme 21:**
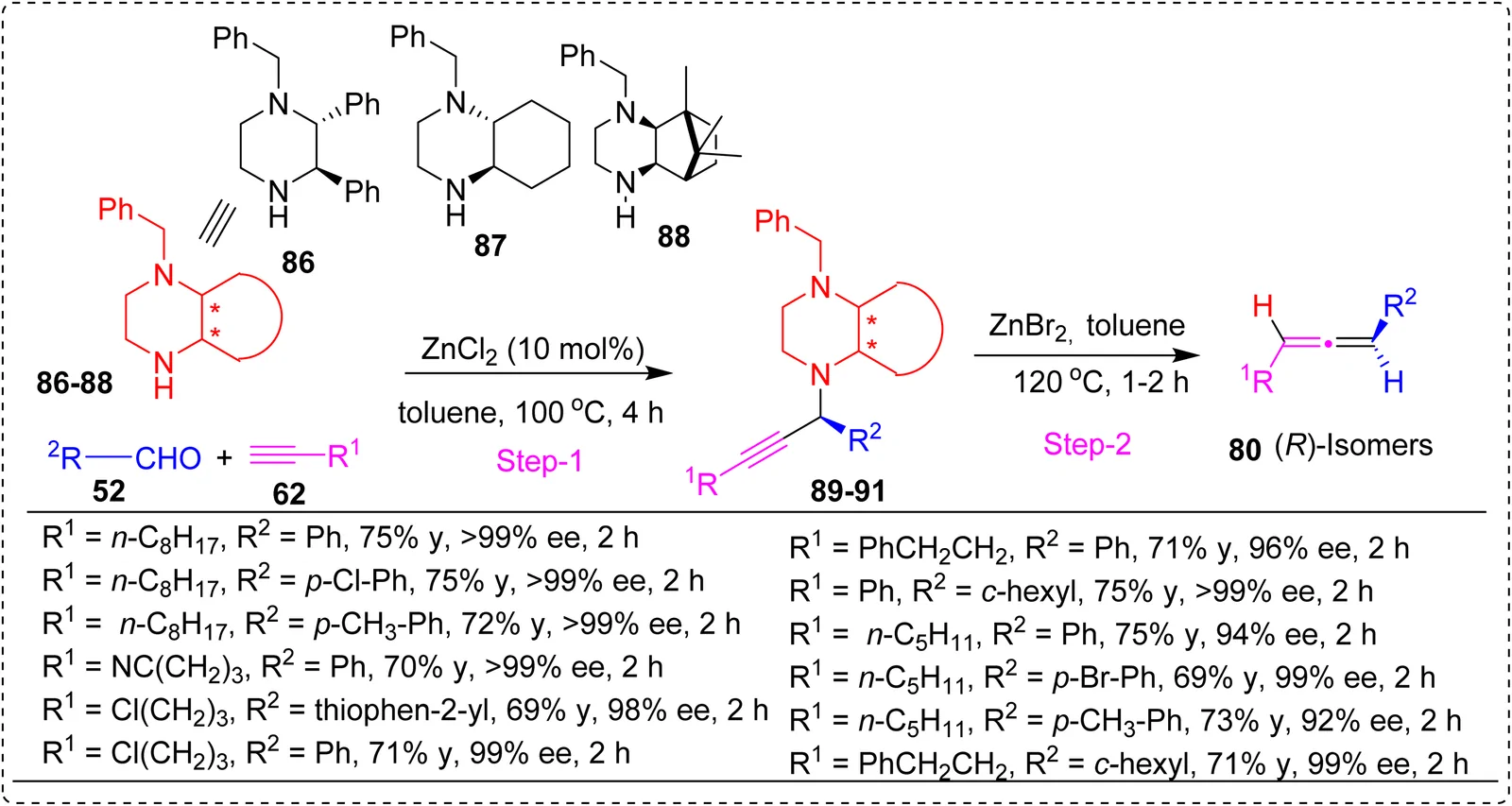
Chiral-piperazine-mediated propargylamine 89–91 formation and zinc-promoted allenation; recovery requires the reported separate reduction/work-up sequence.^17^

**Scheme 22:**
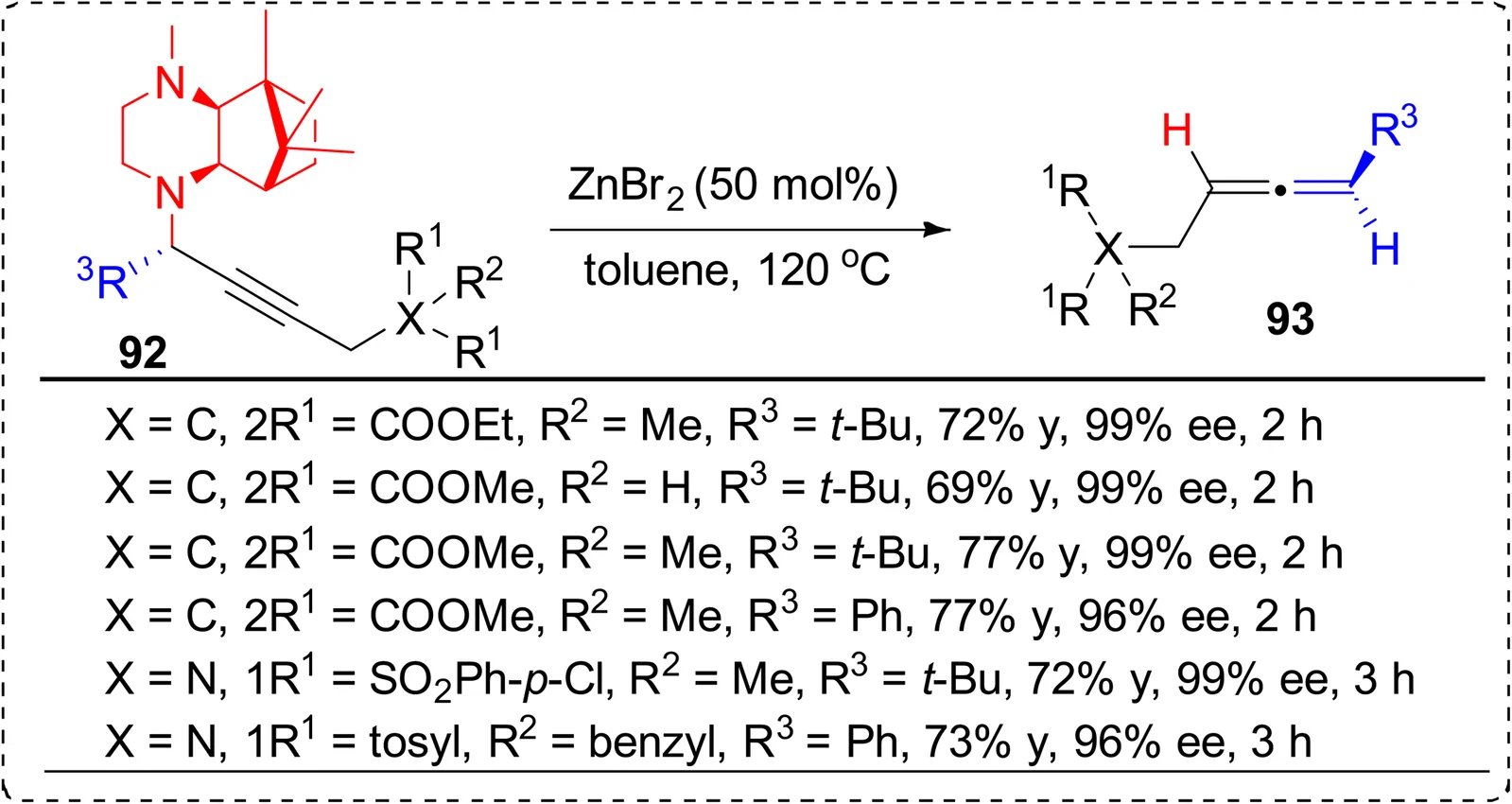
Representative functionalized allenes 93 accessible through chiral-amine-mediated synthesis.^44^

The piperazine studies connect the configuration and diastereomeric composition of a covalent propargylamine 92 with the configuration and ee of the resulting allene 93.^17,43,44^ Conformational restriction and differentiation of the iminium environment offer a rationale for the template effects, but a proposed steric model is not by itself evidence for one universal transition-state geometry across the different scaffolds.

Practical assessment of the piperazine platform must include mediator preparation, rearrangement temperature, functional-group compatibility and the reagent and work-up costs of reductive regeneration.^17,43,44^ Section 6 considers these requirements alongside yield and stereoselectivity.

### 5.2 Pyrrolidine, morpholine, and related cyclic amines

Periasamy and co-workers used optically active 2-benzylmorpholine 94 and *N*-methylcamphanyl 95 piperazine in CuCl-promoted hydroamination/metalation chemistry of terminal alkynes 62.^45^ The resulting diastereomerically enriched, tetrasubstituted propargylamines underwent ZnBr_2_-promoted conversion into trisubstituted chiral allenes 99, with reported allenation yields of 71–89% and enantioselectivities up to 99% ee. The sequence combines an alkyne-derived iminium partner with an alkynyl nucleophile to assemble the rearrangement precursor (Scheme 23).

**Scheme 23:**
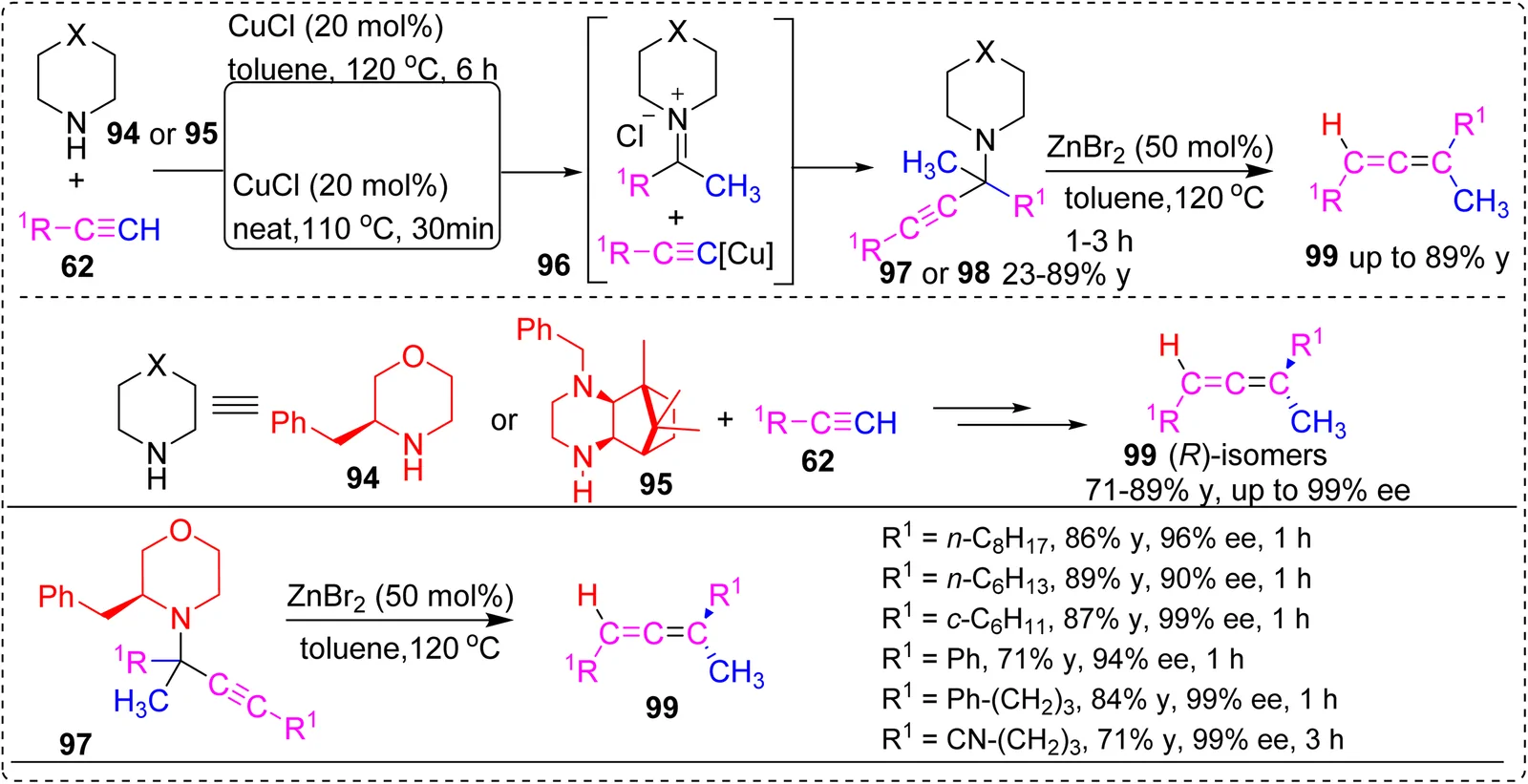
Chiral-amine-mediated hydroamination/metalation and rearrangement sequence giving trisubstituted allenes 99.^45^

This route extends the covalent-relay approach beyond 1,3-disubstituted allenes 99.^45^ Its efficiency depends on the amine scaffold, alkyne substitution and high-temperature precursor-formation and rearrangement steps. The reported ee values establish the enantiomeric composition of the isolated products, but do not determine their racemization barriers under other conditions.

Related 2-dialkylaminomethylpyrrolidines were used by Gurubrahamam and Periasamy to prepare chiral propargylamines from aldehydes and terminal alkynes, followed by copper-promoted conversion into allenes.^46^ This separate mediator family links diastereoselective precursor formation with subsequent allene ee and highlights the need to evaluate both steps when comparing amine structures.

### 5.3 β-Allenoates

Periasamy and co-workers combined DPPE catalysis and ZnI_2_ promotion with a chiral prolinol 63b, terminal alkynes 62 and propiolates 100 to obtain enantioenriched β-allenoates 102 through oxazolidine/iminium and propargylamine intermediates.^47^ The reported β-allenoates contain a methylene group between the ester carbonyl and the allene; they are not conjugated 2,3-allenoates. The nonconjugated ester provides a handle for hydrolysis, reduction and further derivatization (Scheme 24).

**Scheme 24:**
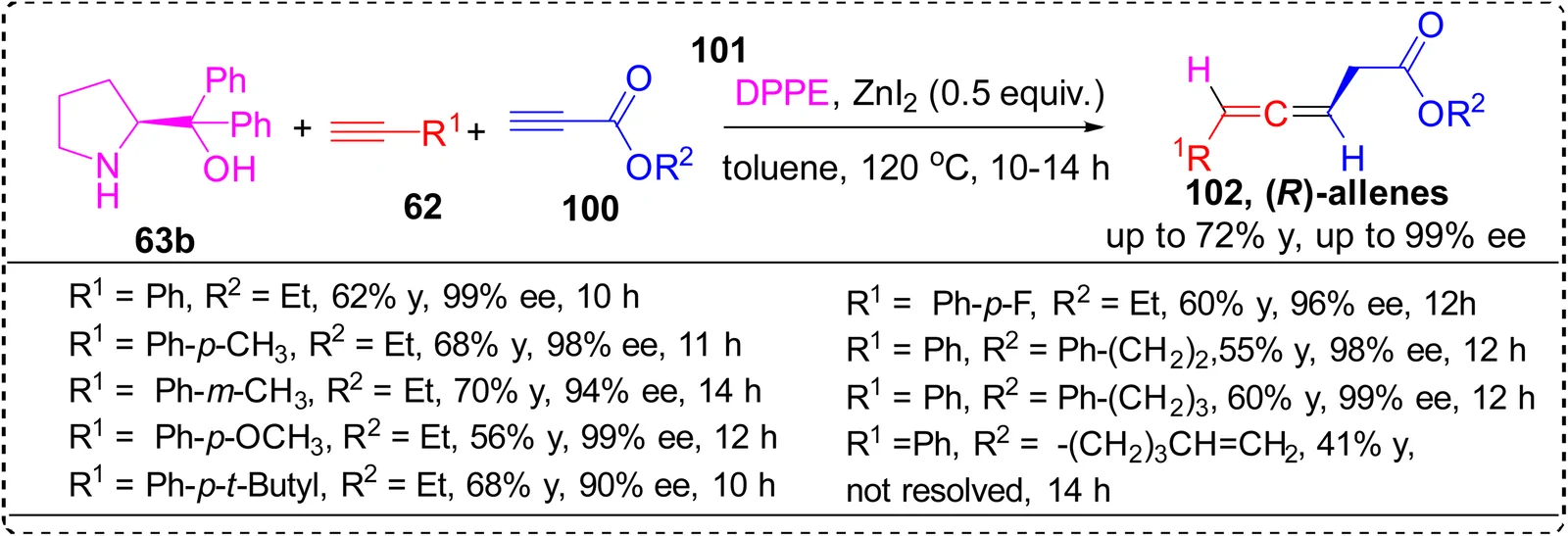
DPPE-catalyzed, ZnI_2_-promoted synthesis of nonconjugated β-allenoates 102 using a stoichiometric chiral amine 63b.^47^

The isolated propargylamine intermediate was converted independently into the corresponding β-allenoate, supporting its role in the reaction sequence.^47,48^ DPPE and ZnI_2_ facilitate precursor assembly and subsequent conversion, while the stoichiometric chiral amine supplies stereochemical information. The transformation therefore represents phosphine-catalyzed, zinc-promoted covalent mediation.

### 5.4 Decahydroquinoxaline scaffolds

Alakonda and Periasamy examined decahydroquinoxaline-derived chiral amines 103 for one-pot allenation of terminal alkynes 62 and aromatic aldehydes 52 with ZnI_2_ in toluene at 120 °C.^19^ The optimized mono-*N*-benzyl mediator derives from a C_2_-symmetric parent scaffold but is not itself C_2_-symmetric. The results section reports 4 h, with yields of 57–85% for aliphatic alkynes, 25% for the aromatic-alkyne example and measured enantioselectivities of 83–96% ee (Scheme 25).

**Scheme 25:**
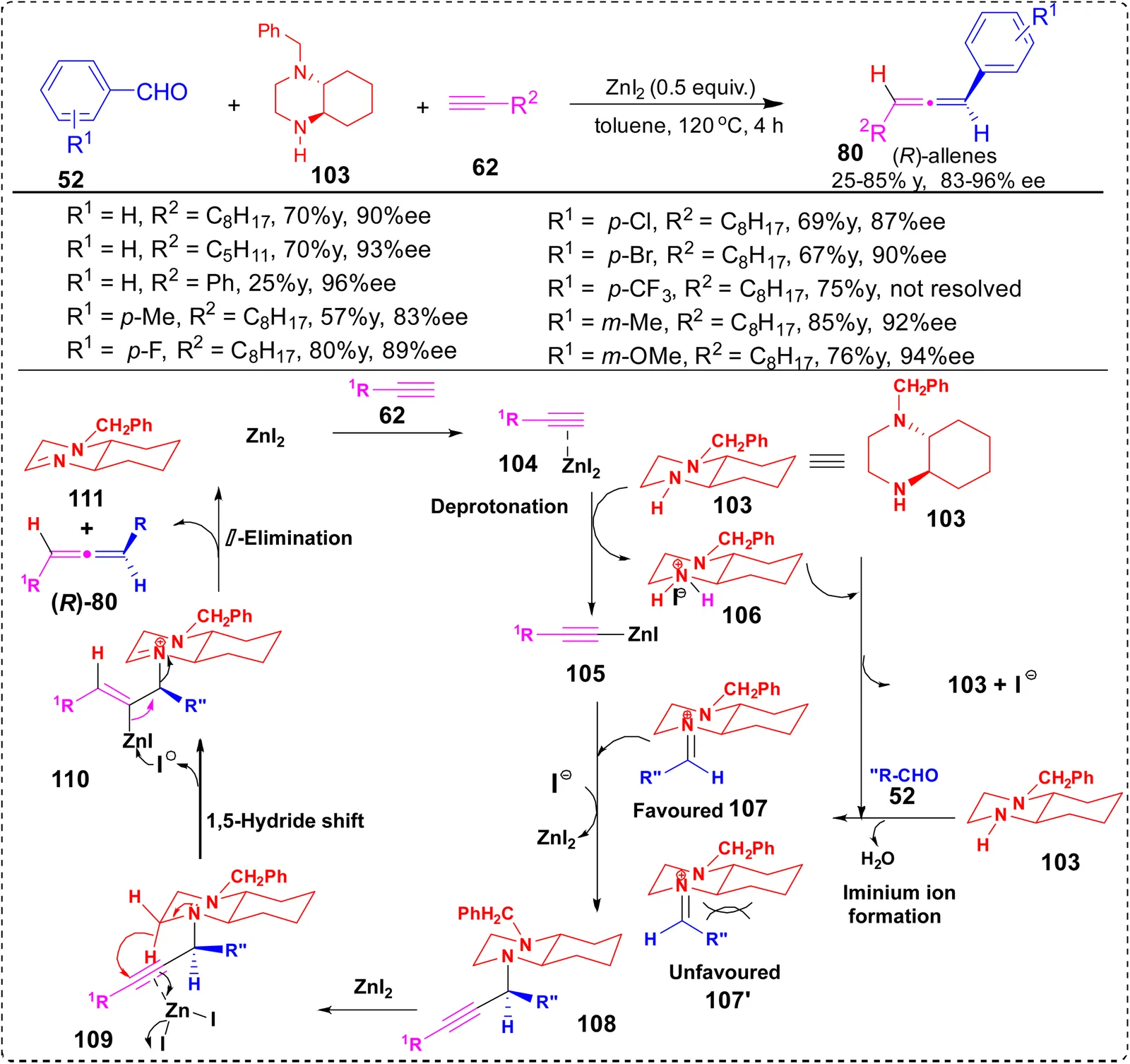
ZnI_2_-promoted one-pot allenation using a mono-*N*-benzyl decahydroquinoxaline-derived mediator 103. The reaction and aldehyde addition were conducted at 120 °C, with a reaction time of 4 h, as clarified by the corresponding author of ref. 19.^19^

The author of ref. 19 has confirmed that the reaction was performed at 120 °C for 4 h, with aldehyde addition also at 120 °C. The 12 h stated in the general procedure and 130 °C stated in the optimization-table footnote are typographical errors. Direct amine recovery from the one-pot mixture was not achieved under the conditions examined. In a separate sequence, an isolated propargylamine was rearranged and the imine-containing mixture treated with NaBH_4_/MeOH to afford 75% regenerated amine. This result demonstrates chemical regeneration and isolated recovery for that sequence, without demonstrating recovery from the direct three-component reaction or multiple productive reuse cycles.

Mono-*N*-benzylation improved the reported outcome relative to the parent diamine, for which bis-propargylamine-derived species were considered a competing pathway.^19^ This scaffold effect supports controlled stoichiometric mediation; it does not establish which elementary event controls stereoselectivity or demonstrate a closed amine-catalytic cycle.

## 6 Comparative analysis of the available strategies

Table 1 collects selected reaction benchmarks, not a ranked comparison of synthetic methods. Each row identifies a matched experiment or a scope summary and retains the stated denominator and yield basis where available. A maximum yield and a maximum ee from different substrates are not paired. Single-step yields, yields based on conversion, and combined multistep yields remain distinct. Differences in substrate activation, substitution pattern, catalyst or mediator amount, scale and reaction time preclude claims of direct superiority from these values alone. Where the reporting basis remains unspecified, the number is retained as a reported yield without assuming that it is isolated. The table therefore supports comparison within a reaction family and identification of practical trade-offs across families.

The comparison reveals complementary capabilities. Activated substrates enable catalytic axis construction and, in some cases, simultaneous axial and central stereocontrol. Covalent propargylamine relays offer modular alkyne/carbonyl combinations but add mediator preparation or regeneration requirements. An amine used at 10–20 mol% and an amine used stoichiometrically impose different material demands even when their product ee values are similar. Preparative demonstrations establish feasibility for the specified examples, rather than uniform scalability. Recovery and reuse are evaluated separately from product yield, as defined in Section 2.2.

### 6.1 Stereochemical efficiency and proof

Configuration assignment and evidence for stereochemical origin should be read separately. Zhou *et al.* assigned source product 3ac by single-crystal X-ray analysis, while dr and ee were measured by ^1^H NMR and chiral HPLC, respectively.^24^ Ji *et al.* provide crystallographic data for allenamide 3a (CCDC 2499680), alongside chiral-HPLC analysis.^26^ These crystallographic results provide reference assignments for the corresponding product series. Assignments in other allene families require independent experimental support. For products assigned by analogy, the relationship to the directly characterized compound must be stated. Chiral chromatography establishes enantiomeric composition, while optical rotation alone is vulnerable to differences in solvent, concentration, purity and calibration.

Diastereomeric ratios of propargylamine intermediates should be connected explicitly to allene ee. For an ideal two-diastereomer mixture, a 99 : 1 precursor dr predicts 98% ee only if the two diastereomers undergo complete, equally efficient, and stereospecific conversion to opposite allene enantiomers: ee = |99 − 1|/(99 + 1) × 100 = 98%. Unequal conversion, epimerization, racemization, selective decomposition, or non-stereospecific rearrangement invalidates that prediction. A high precursor dr followed by lower product ee can therefore reveal loss of stereochemical fidelity, whereas an apparently higher product ee may reflect kinetic enrichment or purification rather than perfect transfer.

The strength of pathway evidence also varies by system. Conversion of an isolated propargylamine into an allene directly supports precursor competence,^13–17,47,48^ but does not by itself establish exclusive flux through that intermediate. The silver study includes a product-racemization control,^14^ whereas the Wolff-rearrangement study shows that a propargylamide can isomerize to an enantioenriched allene and that racemic allenamide is recovered without enantioenrichment under the tested catalyst conditions.^26^ The latter control argues against simple resolution of the allene in that experiment; it is not a racemization test on an enantioenriched allene. None of these observations substitutes for a complete precursor/product balance. Where selective decomposition, precursor epimerization or product racemization has not been assessed in the evidence summarized, these alternatives remain unresolved.

### 6.2 Step economy scale and sustainability

One-pot operation can reduce intermediate isolation, but solvent and material savings depend on the complete procedure. Zinc-halide demand, chiral-amine equivalents, drying agents, chromatography, metal removal and any reductive regeneration contribute to the overall resource requirement. Reaction mass efficiency, process mass intensity and the recovered mediator fraction would allow more informative comparisons than yield and step count alone.

Preparative demonstrations already include the independently checked 100 mmol α-allenol procedure, the 10 mmol dimethylprolinol-mediated example and a 3.5 mmol organocatalytic allenamide preparation.^23,38,40^ These examples establish feasibility for particular substrates; they do not establish uniform scalability across a reported scope. Broader assessment requires isolated yield and ee at increased scale together with reaction concentration, mixing, work-up and thermal conditions, especially for sealed operations at 110–130 °C.

### 6.3 Relationship to foundational and non-amine allene synthesis

The classical Crabbé homologation converted terminal alkynes into allenes using formaldehyde and an amine.^49^ This foundational transformation established the alkyne–formaldehyde–amine combination from which subsequent carbonyl-substrate extensions and asymmetric covalent-relay variants developed.

Modern catalytic routes provide useful benchmarks for evaluating what is distinctive about chiral-amine control. Copper-catalyzed enantioselective proton migration of skipped enynes constructs allenes without a covalent amine relay,^50^ whereas organocatalytic conversion of racemic propargylic alcohols and asymmetric 1,8-addition to aza-para-quinone methides provide highly substituted chiral allenes through non-amine stereocontrol.^51,52^ Chiral-ligand-controlled copper catalysis offers another mechanistically distinct route to axially chiral allenes.^53^ These methods fall outside the central scope of this review, but they define important standards for catalyst loading, substrate generality, temperature, and directness against which chiral-amine-enabled approaches should be compared.

The stereochemical foundation of this chemistry predates modern asymmetric catalysis. Maitland and Mills provided an early experimental demonstration of allene asymmetry in 1935.^54^ Its inclusion here emphasizes that current synthetic advances address a long-standing stereochemical problem: controlling the configuration of an axis generated during bond reorganization.

## 7 Synthetic applications of chiral allenes obtained through amine control

### 7.1 Carbocyclic synthesis and axial-to-central chirality transfer

The cyclohexenylallene/Yb(OTf)_3_ sequence illustrates the conversion of allenic axial information into central chirality through [4+2] cycloaddition.^42^ The resulting carbocyclic adducts 114/115 demonstrate the synthetic value of the conjugated allene architecture (Scheme 26). Their ee reflects the combined outcome of asymmetric allenation and cycloaddition; quantifying the fidelity of chirality transfer additionally requires the ee of the corresponding allene input.

**Scheme 26:**
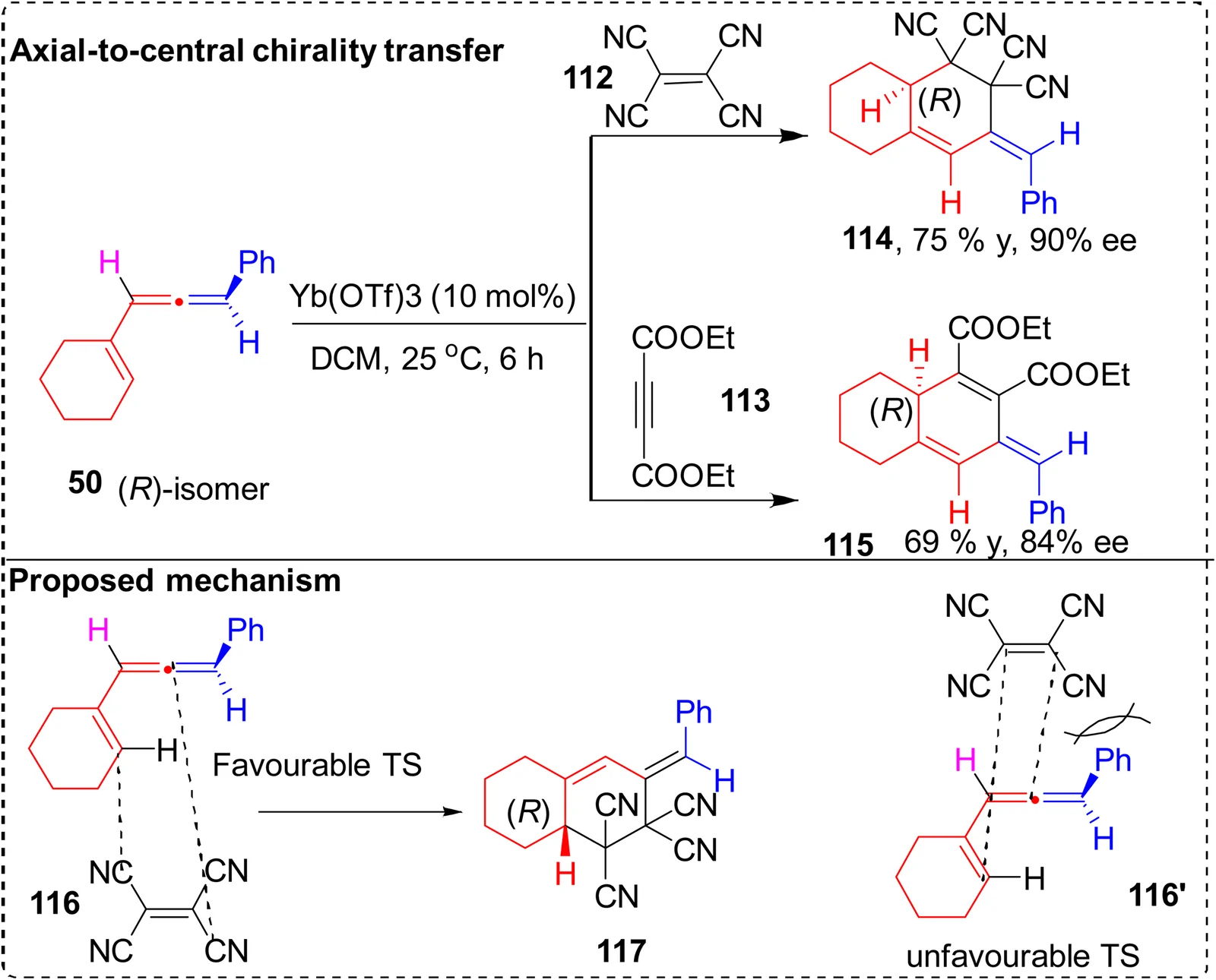
Yb(OTf)_3_-catalyzed cycloaddition of cyclohexenylallenes 50 and axial-to-central chirality transfer.^42^

### 7.2 Natural products and functionalized building blocks

Periasamy and co-workers applied chiral-amine-mediated allenation to oxygenated building blocks relevant to natural-product 120 and insect-pheromone 123 synthesis.^44^ These applications include an allenic metabolite associated with *Sapium japonicum* and functionalized allene intermediates for further elaboration toward pheromone targets (Scheme 27). They demonstrate access to synthetically useful functionality alongside the allene axis.

**Scheme 27:**
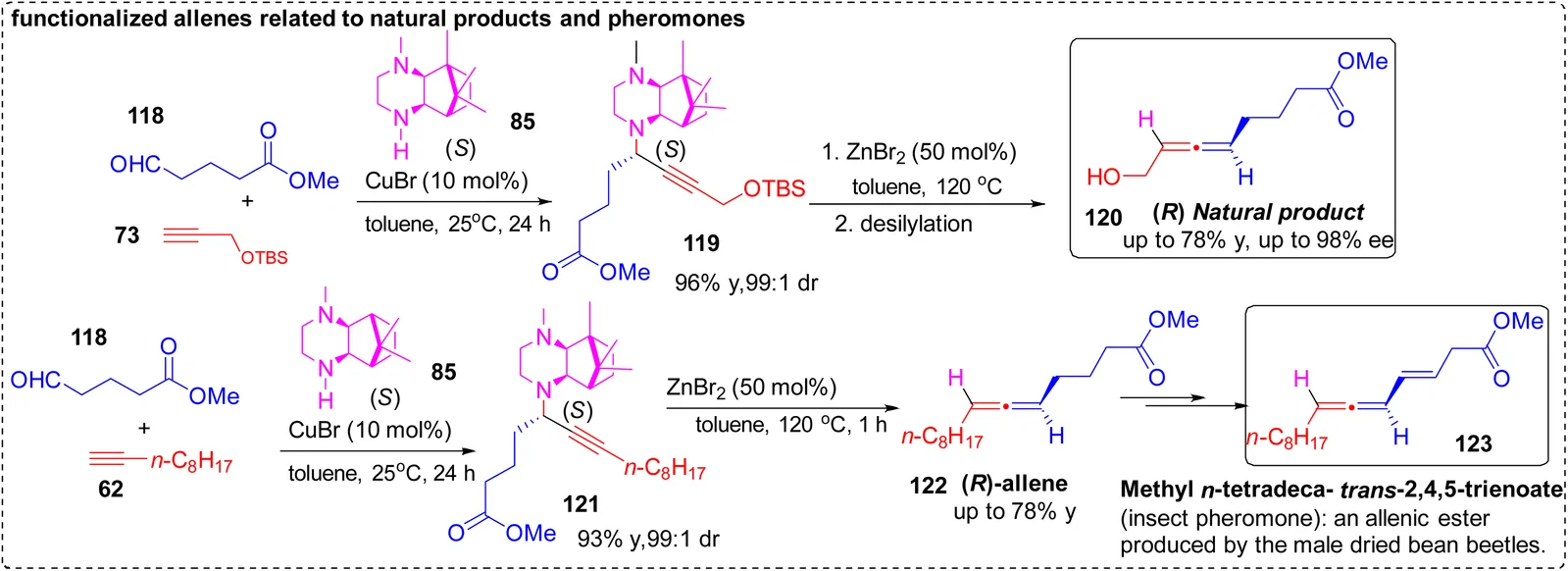
Functionalized allene products and intermediates related to natural-product and pheromone synthesis.^44^

Tang and co-workers broadened the CuBr_2_/diphenylprolinol strategy to allenes bearing unprotected functional groups, including naturally occurring targets.^55^ Their synthesis of laballenic acid exemplifies direct construction of a functionalized allene from a terminal alkyne and an aldehyde, with configuration controlled by the prolinol enantiomer. The demonstrated functional-group compatibility extends the synthetic scope of the approach, although stoichiometric mediator use and elevated-temperature operation remain practical constraints.

Allenols 124 provide opportunities for diversification while retaining the allene unit. The prolinol/CuBr_2_ study demonstrated oxidation to aldehydes 125 and Mitsunobu substitution with phthalimide 127.^38^ Vinylallenes 50 offer a complementary mode of reactivity: their (hetero-Diels-Alder reactions consume the allene axis while generating central chirality (131 & 132).^15^Scheme 28 contrasts functional-group modification around a retained axis with conversion of axial information into new stereogenic centres.

**Scheme 28:**
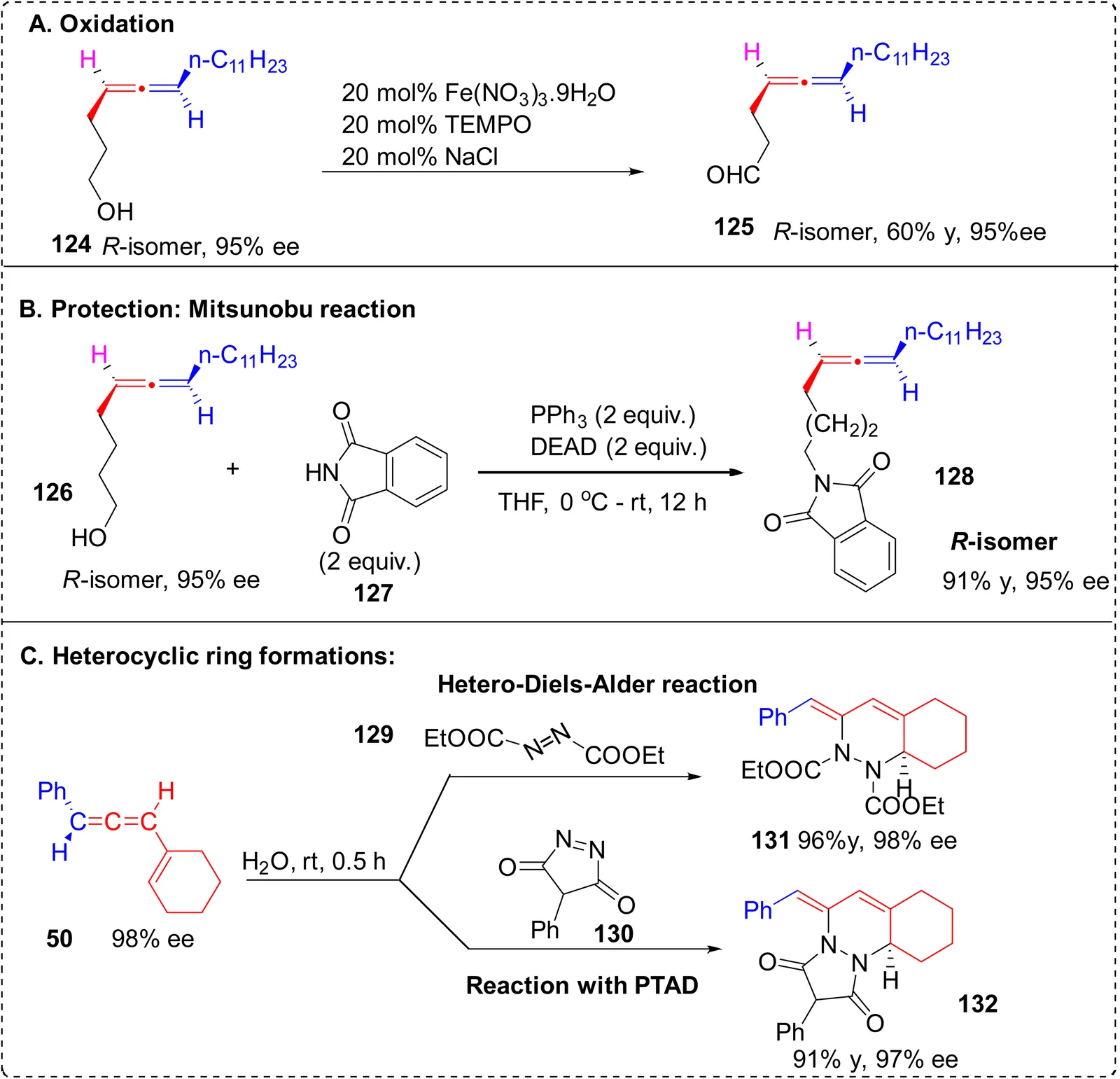
Allenol 124 oxidation (A, 125),^38^ and Mitsunobu phthalimidation (B, 128),^38^ and vinylallene 50 cycloaddition with axial-to-central chirality transfer (C, 131 & 132).^15^

## 8 Mechanistic evidence and unresolved questions

### 8.1 What is established

The strongest evidence establishes specific transformations rather than a universal mechanism: independently prepared propargylamines form allenes,^13–17^ the piperazine studies connect precursor stereochemistry with product configuration,^17,43^ and the dimethylprolinol/diphenylprolinol comparison measures scaffold effects within matched substrate pairs.^38^ Copper/zinc control experiments show that both metals affect the bimetallic reaction.^36^ These observations establish precursor competence or help define metal function. Further pathway-specific evidence would help distinguish the proposed hydrogen-transfer steps, metal resting states and stereochemistry-determining transition structures.

The evidence is less uniform regarding the exact transition state and the resting state of the metal. A density-functional study of Ag(i)-catalyzed conversion of propargylamines to axially chiral allenes proposed coordination of silver anti to nitrogen, followed by rate-limiting hydride transfer and anti-periplanar elimination.^18^ This analysis provides a chemically explicit alternative to a generalized concerted 1,5-hydrogen-shift description, although its conclusions should not be transferred automatically to copper- or zinc-promoted systems. Copper may participate in acetylide formation, iminium addition, or subsequent rearrangement, while zinc may coordinate nitrogen, activate the C–N bond, or stabilize a hydrogen-transfer geometry. Different substrates and metals may shift the balance among these roles; a single universal mechanistic drawing is therefore unlikely to describe every protocol.

### 8.2 Experiments needed for stronger mechanistic conclusions

Future studies would benefit from kinetic order measurements in amine, metal, aldehyde, and alkyne; direct observation of intermediates by *in situ* NMR or IR spectroscopy; isotope effects and position-specific deuterium labeling; crossover experiments; and independent reactions of each propargylamine diastereomer. Competition experiments can reveal which structural feature controls rate rather than selectivity.

Computational analysis should include conformational sampling and alternative protonation or metal-coordination states. Analysis based on a limited number of proposed transition structures may not capture the full conformational and coordination landscape. Calculated barriers should be evaluated against observed temperature and rate, and predicted configurations should be tested across substrates that change steric and electronic effects independently.

Nonlinear effects and catalyst ee studies would be informative for truly catalytic amine systems. A linear product-ee/catalyst-ee relationship is consistent with, but does not prove, a monomeric stereochemistry-determining catalyst. Positive or negative nonlinear effects may indicate catalyst aggregation, reservoir species, or competing background pathways.

## 9 Outlook

The next advance for modular covalent relays is to couple selective precursor formation with regeneration of the active secondary amine under productive reaction conditions. The definitions in Section 2.2 separate this objective from post-reaction recovery. A useful demonstration would quantify product formation per initial amine amount, identify the regenerated species, and preserve configuration and material balance over successive turnovers.

Temperature remains an important limitation of many covalent-relay protocols, which commonly operate at 110–130 °C. By comparison, several activated-substrate organocatalytic and cooperative reactions proceed at lower temperatures, although they use different substrate classes (Table 1). New thermal, photochemical or electrochemical activation strategies should therefore be assessed within matched reaction families and tested for product racemization as well as conversion.

Scope expansion must also be defined for individual methods. Noncovalent catalysis often relies on an ester, ketone, imino ester or organizing amide; broader access from less activated substrates remains desirable. Covalent-relay methods face different constraints, including iminium formation, acetylide addition and rearrangement. Testing α-branched or heteroaromatic aldehydes, enals, aromatic alkynes and multifunctional substrates is informative only when failures are assigned to the responsible step. Negative results and quantitative balances of intermediates would make these limitations easier to address.

Independent control of axial and central chirality is already demonstrated in protected secondary-allenol synthesis and in cooperative palladium/primary-amine allenylation.^33,39^ The latter gives access to all four stereoisomers through catalyst combinations, with selectivity depending on the substrate and catalyst pairing. Further progress should extend this control across product classes, improve challenging diastereomeric combinations and retain practical catalyst loading and isolation efficiency.

Comparable reporting should place loading, yield basis, concentration, temperature, time, scale, ee or dr, configuration assignment and product stability alongside one another. Where a mediator is regenerated, the reduction/work-up, recovery denominator and performance in successive reuse experiments should be reported explicitly. Linking these measurements would make mechanistic interpretation and preparative selection more reliable.

## 10 Conclusion

The value of chiral amines in allene synthesis lies in their adaptable operational roles. Catalytic noncovalent and covalent activation can construct or functionalize an allenic axis, including products with additional stereogenic centers, while stoichiometric propargylamine relays connect modular alkyne and carbonyl building blocks to central-to-axial chirality transfer. A useful choice between these routes therefore starts with the required substitution pattern and available substrate activation, then considers the complete reagent and regeneration balance. High ee alone cannot resolve that choice or establish a mechanism. The integrated comparison identifies two priorities: stronger experimental links between precursor configuration and product outcome, and regeneration strategies that preserve the modularity of covalent relays while reducing fresh amine demand. Substrate-matched studies of lower-temperature activation, configurational stability and successive mediator reuse would connect mechanistic progress with preparative value. Until such data are available, catalytic turnover, post-reaction recovery and mechanistic inference should remain explicitly separate claims.

## Conflicts of interest

The authors declare no competing financial interest.

## Data Availability

No primary research results, software or code have been included, and no new data were generated or analysed as part of this review.
